# Biological and Physico-Chemical Characteristics of Arginine-Rich Peptide Gemini Surfactants with Lysine and Cystine Spacers

**DOI:** 10.3390/ijms22073299

**Published:** 2021-03-24

**Authors:** Damian Neubauer, Maciej Jaśkiewicz, Marta Bauer, Agata Olejniczak-Kęder, Emilia Sikorska, Karol Sikora, Wojciech Kamysz

**Affiliations:** 1 Department of Inorganic Chemistry, Faculty of Pharmacy, Medical University of Gdańsk, 80-416 Gdańsk, Poland; mj@gumed.edu.pl (M.J.); marta.bauer@gumed.edu.pl (M.B.); karol.sikora@gumed.edu.pl (K.S.); wojciech.kamysz@gumed.edu.pl (W.K.); 2 Department of Histology, Faculty of Medicine, Medical University of Gdańsk, 80-211 Gdańsk, Poland; agata.olejniczak@gumed.edu.pl; 3 Department of Organic Chemistry, Faculty of Chemistry, University of Gdańsk, 80-308 Gdańsk, Poland; emilia.sikorska@ug.edu.pl

**Keywords:** cationic lipopeptides, antimicrobial lipopeptides, gemini surfactants, gemini lipopeptides, arginine-rich lipopeptides, ESKAPE, antifungal, antibacterial, antibiofilm

## Abstract

Ultrashort cationic lipopeptides (USCLs) and gemini cationic surfactants are classes of potent antimicrobials. Our recent study has shown that the branching and shortening of the fatty acids chains with the simultaneous addition of a hydrophobic *N*-terminal amino acid in USCLs result in compounds with enhanced selectivity. Here, this approach was introduced into arginine-rich gemini cationic surfactants. l-cystine diamide and l-lysine amide linkers were used as spacers. Antimicrobial activity against planktonic and biofilm cultures of ESKAPE (*Enterococcus faecium*, *Staphylococcus aureus*, *Klebsiella pneumoniae*, *Acinetobacter baumannii*, *Pseudomonas aeruginosa*, and *Enterobacter* spp.) strains and *Candida* sp. as well as hemolytic and cytotoxic activities were examined. Moreover, antimicrobial activity in the presence of human serum and the ability to form micelles were evaluated. Membrane permeabilization study, serum stability assay, and molecular dynamics were performed. Generally, critical aggregation concentration was linearly correlated with hydrophobicity. Gemini surfactants were more active than the parent USCLs, and they turned out to be selective antimicrobial agents with relatively low hemolytic and cytotoxic activities. Geminis with the l-cystine diamide spacer seem to be less cytotoxic than their l-lysine amide counterparts, but they exhibited lower antibiofilm and antimicrobial activities in serum. In some cases, geminis with branched fatty acid chains and *N*-terminal hydrophobic amino acid resides exhibited enhanced selectivity to pathogens over human cells.

## 1. Introduction

**Figure 1:**
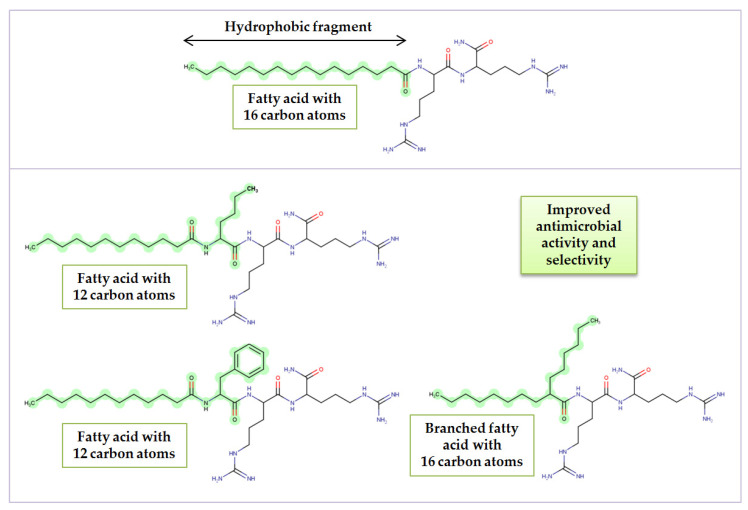
Effect of *N*-terminal amino acid and fatty acid type on antimicrobial activity and selectivity.

Lipopeptides are a class of compounds consisting of amino acids linked by amide bond with at least one lipid chain attached. Lipopeptides are amphiphilic owing to a peptide fragment that is hydrophilic and a fatty acid residue as a hydrophobic part. They can be both biosynthesized by microorganisms or obtained through chemical synthesis. In fact, the conjugation of a fatty acid chain to a peptide was found to be a convenient tool for obtaining molecules with improved antimicrobial activity [1]. A promising group of compounds are ultrashort cationic lipopeptides (USCLs). They are relatively small (up to seven amino acid residues) and are potent antimicrobial agents. Usually, antimicrobial peptides (AMPs) and USCLs are cationic species facilitating electrostatic interactions with negatively charged pathogenic cell surface. Lipopeptide molecules disrupt the cell membrane, participate in its permeabilization, and cause cell death. Lipopeptide antibiotics found in nature (daptomycin, echinocandins, polymyxins) turned out to be very potent drugs against pathogens and have successfully been applied for the treatment of life-threating infections. In 2017, in the *Essential Medicines List*, published by the World Health Organization (WHO), daptomycin and polymyxins were classified as reserve antibiotics or so-called “last resort” (status maintained in the newest database, 2019) [2,3]. Polymyxins are used to combat resistant Gram-negative pathogens such as carbapenem-resistant *Pseudomonas aeruginosa*, *Acinetobacter baumannii*, and *Enterobacteriaceae*, while daptomycin is used to treat infections caused by resistant Gram-positive strains, e.g., methicillin-resistant *Staphylococcus aureus* (MRSA) and vancomycin-resistant enterococci (VRE) [4,5]. Unfortunately, polymyxins can cause nephrotoxicity and neurotoxicity. On the other hand, at times, polymyxins are the only drugs that can be used to treat critically ill patients with multidrug resistant infections [6]. Treatment with daptomycin is associated with rhabdomyolysis and an increase in creatine phosphokinase (CPK) [7,8]. According to the latest epidemiological reports, resistance to these clinically used lipopeptide drugs is still relatively rare, but it cannot be ignored in view of a noticeable tendency of increasing rate of resistance and “drug of last resort” status [4,9,10,11]. In response to this global issue, in 2017, the WHO, for the first time, published a priority pathogen list, named ESKAPE strains (*Enterococcus faecium*, *Staphylococcus aureus*, *Klebsiella pneumoniae*, *Acinetobacter baumannii*, *Pseudomonas aeruginosa*, and *Enterobacter* spp.), that urgently need new antibiotics [12]. Moreover, the 2020 update warned that only a few new antibacterial treatments are in development [13]. In general, antimicrobial peptides exhibit a low level of induced resistance owing to their mechanisms of action [14]. Nevertheless, pathogens are still able to create some response to protect themselves from AMPs. In our previous study, we demonstrated that shortening of the fatty acid chain with the simultaneous addition of a hydrophobic amino acid residue at the *N*-terminus resulted in more selective and active compounds than those of the reference lipopeptides of comparable lipophilicity. USCLs with branched fatty acid chains gave similar results [15]. This concept is presented in Figure 1.

**Figure 2:**
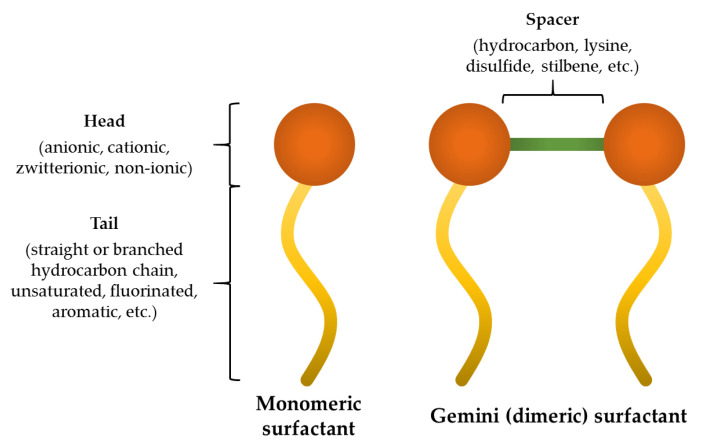
General structure of monomeric and dimeric (gemini) surfactants.

Another interesting group of molecules are gemini surfactants (dimeric surfactants). In general, those surfactants contain two hydrocarbon tails and two hydrophilic heads linked at or near the head-groups by a spacer (Figure 2).

The efforts of scientists resulted in obtaining gemini surfactants with different head groups that can be anionic (e.g., alkylbenzene sulfonate, phosphate, dicarboxylate) [16,17,18], cationic (e.g., quaternary ammonium, arginine, lysine, serine, imidazolium, morpholinium, pyridinium, piperidinium, pyrrolidinium) [19,20,21,22,23,24,25], zwitterionic (e.g., betaine, heterogemini surfactants) [26,27], and non-ionic (e.g., 3-deoxy-D-glucitol, lactobionic acid residues) [28,29]. Moreover, plenty of different spacers were used such as hydrocarbons (saturated and unsaturated), stilbene units, p-xylenes, cyclohexane derivatives, disulfides, lysine esters, triazines, or even borates [30,31,32,33,34,35]. Formally, geminis that are asymmetric (e.g., those with a lysine spacer) are classified as geminoid or gemini-like surfactans [36,37]. Hydrophobic fragments (tails) used in gemini surfactants vary in length (usually 8–20), structure (straight or branched chain) [38], and saturation [39]; tails can be fluorinated [40], contain poly(ethylene oxide) [41], cholesterol [42], or aromatic backbones [43]. Gemini surfactants are an intriguing group of compounds owing to their increased solubility in water, superior surface tension reduction power, reduced proximity between hydrophobic groups, lower critical micellar concentration (by one or more magnitudes), and superior wetting characteristics as compared to the corresponding single chain (monomeric) surfactants, which makes them convenient candidates for pharmaceuticals formulations [44]. Dimeric surfactants revealed to be potent gene-delivery carriers (transfection agents for non-viral gene therapy) and also drug delivery carriers as additives in liposome formulations [36,45]. It has been shown that dimeric surfactants exhibited improved antimicrobial activities against a broad spectrum of microorganisms as compared to those of the monomeric surfactants [46].

**Figure 3:**
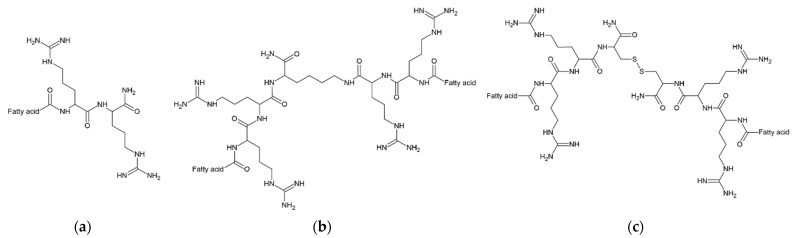
General structures of lipopeptides: (**a**) USCL—parent molecules; (**b**) gemini lipopeptides with l-lysine amide spacer; (**c**) gemini lipopeptides with l-cystine diamide spacer. Compounds **B5**, **B6**, **C5**, and **C6** have N-terminal phenylalanine (Phe) or norleucine (Nle) residues, while **B7** and **C7** have 2-butyloctanoic acid residues denoted as C8(4). Structures of **B5–B7** and **C5–C7** are attached as Supplementary Materials, Figures S1–S6. USCL: ultrashort cationic lipopeptides.

This study focuses on cationic gemini surfactants rich in arginine residues. The compounds used in this study are derivatives (dimers) of USCLs studied in the previous research [15]. Dimeric surfactants consist only of amino acids and straight or branched fatty acid chains. The aim of this study was to evaluate the impact of hydrocarbon chain length and structure (straight and branched fatty acids), as well as of spacer (l-cystine diamide and l-lysine amide) on physico-chemical and biological properties. To do so, antimicrobial activities, hemolysis, cytotoxicity (HaCaT cells), hydrophobicity (retention time), and critical aggregation concentrations (CACs) were measured. Antimicrobial activity was determined against ESKAPE strains and yeasts, *Candida albicans* and *Candida glabrata*. Moreover, molecular dynamics, membrane permeabilization kinetics and human serum stability studies were performed on selected compounds. Dimeric surfactants were compared to monomeric parent molecules. General chemical structures are presented in Figure 3.

## 2. Results and Discussion

### 2.1. Determination of Peptide Hydrophobicity and Critical Aggregation Concentration

The identity of the purified peptides was confirmed with electrospray ionization mass spectrometry (ESI-MS) in positive ion mode. Results of MS analyses and net charge are attached in Supplementary Materials (Table S1). Peptides’ hydrophobicity was evaluated by analytical reversed-phase high-performance liquid chromatography (RP-HPLC). The critical aggregation concentrations (CACs) of the lipopeptides were determined in unbuffered aqueous solution at 298 K by measuring the surface tension as a function of lipopeptide concentration. The measurements were carried out by the Du Noüy ring method on a K100 tensiometer. The surface tension data were plotted against the logarithm of lipopeptide concentration. CAC was found as the intersection of two lines being linear regression of data below and above CAC. Adjusted retention times (**tR’**) and CACs are displayed in Table 1.

In general, the hydrophobicity of gemini surfactants with a cystine spacer was higher than that with a lysine amide spacer (**C** and **B**, Table 1). The only exception was peptides **B4** and **C4** with myristic acid residues. Gemini surfactants with branched fatty acid chains (**B7** and **C7**) were less hydrophobic than the corresponding gemini surfactants with an identical number of carbon atoms in straight-chain fatty acid residues (**B3** and **C3**, respectively). This finding is consistent with our previous conclusions where USCLs with straight-chain fatty acid had elevated hydrophobicity as compared to that of lipopeptides with branched fatty acids [15]. The difference in retention can be explained in terms of a more compact structure and a lower area of the branched compounds that can interact with stationary phase than that of straight-chain ones [47]. In both series, surfactants with phenylalanine residues (**B5** and **C5**) were more hydrophobic than their counterparts with norleucine (**B6** and **C6**). This finding is consistent with those of our previous study where linear ultrashort cationic lipopeptides with N-terminal phenylalanine were more hydrophobic than those with norleucine [15].

The critical aggregation concentration (CAC) values determined by surface tension measurements vs. lipopeptide concentration are collected in Table 1. As expected, the gemini lipopeptides self-assemble at concentrations much lower than those of their monomeric counterparts.

**Figure 4:**
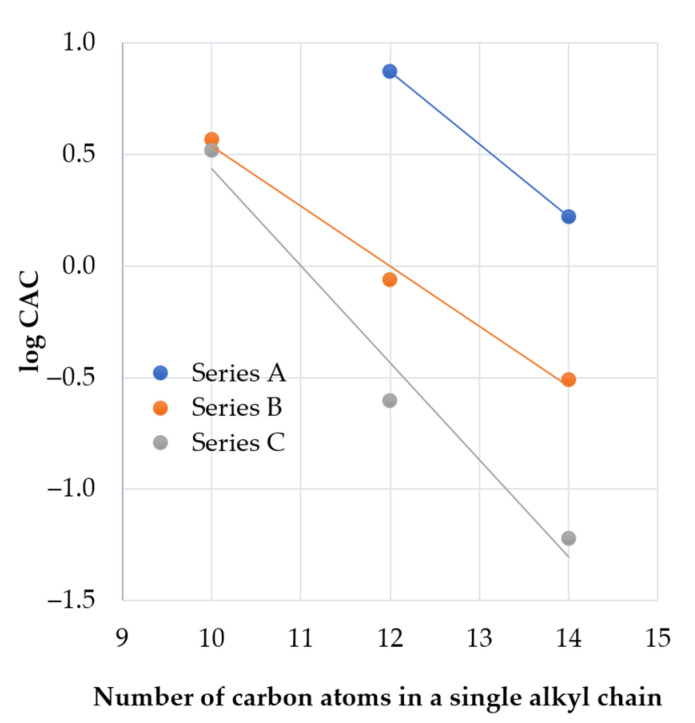
LogCAC vs. carbon atoms in a single hydrophobic chain for each series of the lipopeptides.

In all series of the lipopeptides, an increase in fatty acid chain length results in a decrease of CAC values with a linear dependence between the logarithm of CACs and the number of carbons in a single hydrocarbon chain (Figure 4). This observation is in agreement with the empirical equation found by Klevens for homologous straight-chain ionic surfactants [48]. According to the Klevens equation, LogCMC (or LogCAC) is the linear function of the number of carbon atoms in the chain.

**Figure 5:**
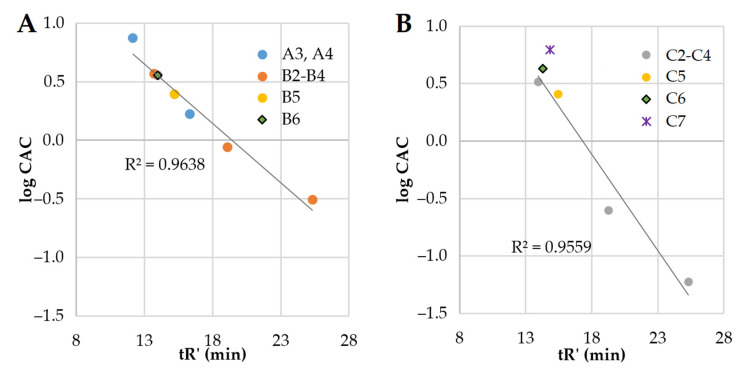
LogCAC vs. adjusted retention time of lipopeptides. (**A**) Series **A** and **B** (compounds **A3**, **A4**, **B2**–**B6**)—linear correlation; (**B**) Series **C** (**C2**–**C7**)—linear correlation (**C7** was excluded).

The elongation of fatty acid chains is correlated with the increasing hydrophobicity of the compound. In this study, branched fatty acids and hydrophobic amino acids were used. To learn how these structural features affect CAC and how it is related to the remaining compounds, we used adjusted retention time (tR’) as an indicator of hydrophobicity. LogCAC vs. tR’ of compounds is plotted in Figure 5.

It can be deduced that the hydrophobicity (tR’) of series **A** and series **B** vs. logCAC is linearly correlated (R^2^ = 0.9638). Similarly, series **C** (excluding compound **C7**) follows that rule (R^2^ = 0.9559). Surfactant **C7** deviates from the linear regression due to its branched fatty acid chains. On the other hand, the retention time and logCAC of surfactants with N-terminal hydrophobic amino acid residues (Phe—**B5** and **C5**, and Nle—**B6** and **C6**) are linearly correlated with the remaining ones (Figure 5). However, the elongation of fatty acid chains more drastically reduces the CAC values in the gemini lipopeptide series with l-cystine diamide as a spacer than in the l-lysine amide ones. Presumably, this phenomenon results from the different flexibility of the spacers, as well as their interactions with aqueous environment. Previous studies on gemini non-peptide cationic surfactants have shown that a greater flexibility of the spacer favors a greater reduction in CAC as the hydrocarbon chains become longer [49]. However, an opposite effect was observed for hydrophilic spacers where CMC was increasing with an elongating ethoxylated spacer [50]. Moreover, a reduction of electrostatic repulsion between headgroups by the hydration around the spacer facilitates the self-assembly and moves the CAC point to lower concentrations [51]. It was found that gemini surfactants with hydrophilic spacers have lower CAC than conventional surfactants [52]. The l-Lysine amide spacer is formally longer than that of the l-cystine diamide (by one atom); however, the disulfide bond is relatively long (≈2.04 Å), which makes these two spacers similar in length [53]. The main differences between the spacers are in their hydrophobicity (analogs with l-cystine diamide are usually more hydrophobic than those with l-lysine, Table 1), flexibility, and the number of hydrogen bond donors and acceptors. l-Lysine amide has only one donor and one acceptor (amide), but l-cystine diamide has two donors and two acceptors (Figure 3). Interactions between amides (hydrogen bonds) can stabilize the micellar structure. It was shown that urea in hydrophobic tails can significantly reduce CMC in comparison to hydrocarbon analogs, owing to hydrogen bonds [54]. Presumably, amide groups in the spacer can also interact with each other and stabilize the micelle, therefore reducing CAC. This can be an explanation of the observed differences between series **B** and **C**.

A comparison of the two gemini lipopeptides, **C3** and **C7**, with the same headgroup, spacer, and the number of carbon atoms in hydrocarbon chains shows that the branching of the acyl chains results in a ca. 25-fold increase in the CAC value. This is due to a steric hindrance affecting the packing of the hydrophobic chains in the micelle core and indicates that branching of the hydrocarbon tail could be in effect equivalent to the chain length reduction. In turn, an introduction of hydrophobic amino acid residues at both extremities of the gemini lipopeptide sequence acts as extension of the acyl chains. Based on the aforementioned relationship between logCAC value and hydrocarbon chain length, it can be concluded that both phenylalanine (Phe) and norleucine (Nle) generate a hydrophobic contribution equivalent to extension of the octyl hydrocarbon chain by another 2–3 methylene groups. Slightly lower CAC values for Phe-containing gemini lipopeptides compared to Nle-containing ones are compatible with an enhanced hydrophobicity of the former (Table 1). In addition, the presence of aromatic moiety is known to facilitate the self-assembly of molecules via π–π stacking interactions [55]. It has been shown that surfactants with branched hydrophobic groups have higher CAC values than the corresponding straight-chain ones [56]. It was stated that branched hydrocarbon chains have weaker intermolecular cohesive forces than those of the straight ones [57]. Moreover, surfactants with bulky hydrophobic groups were shown to have higher CAC than similar but less bulky surfactants due to difficulties with the incorporation of these groups into the micelle [58].

### 2.2. Antimicrobial Activity, Cytotoxicity, and Hemolysis of Lipopeptides

Minimum inhibitory concentrations (MICs) of the test compounds were determined against planktonic cultures of ESKAPE bacterial strains and two *Candida* strains. Hemolysis (HC_50_) and cytotoxicity (IC_50_) to keratinocytes (HaCaT) were examined. Selectivity of compounds over human normal cells were estimated. Selectivity indexes (SIs) were calculated as IC_50_HaCaT/MIC (SI HaCaT) or HC_50_/MIC (SI human red blood cells (hRBCs)). The results are presented in Table 2.

In general, the compounds were the most active against Gram-positive strains (*E. faecium* and both *S. aureus* strains). The majority of compounds exhibited moderate or high antimicrobial activity against both fungal strains. The most resistant strains were Gram-negative bacteria, especially *K. pneumoniae* and *K. aerogenes*. A similar pattern of antimicrobial activities between strains was noticed in the previous study on arginine-rich USCLs [15]. A general statement is that Gram-negative bacteria are less sensitive to cationic lipopeptides than Gram-positive ones, this being consistent with the literature [59,60]. The most hydrophobic and potent monomeric lipopeptide was **A4** (tetradecanoic acid, C14). The antimicrobial activity of gemini surfactants (series **B**, **C**) correlates with fatty acid chain length and hydrophobicity. Similarly, Castillo et al. stated that arginine-based gemini surfactants with decanoic acid have a noticeably higher antimicrobial activity than those with octanoic acid residues (e.g., 2 and 16 µg/mL against *S. aureus* ATCC 25178) [61]. Further studies on this class of compounds revealed that the optimal chain length is C10, which is consistent with our present findings. The HC_50_ of a surfactant with two arginine and decanoic acid residues linked with a 1,3-diaminopropane spacer was found to be 110.5 µg/mL, and MIC against *C. albicans* ATCC 10231 was 16 µg/mL (calculated SI = 6.91) [62]. In this study, geminis with decanoic acid (**B2** and **C2**) are more selective to *C. albicans* ATCC 10231 (SI > 32 vs. 6.91). Furthermore, it was found that optimal number of carbon atoms in straight fatty acid chain is different for bacterial and fungal strains. In the case of bacteria, the optimal length varies between 10 and 12 carbon atoms, while for *Candida* sp., the lowest MIC values were noticed when the straight fatty acid chain contained 12 or 14 carbon atoms. These findings are consistent with previous studies on mono- and diacylated lipopeptides with arginine or lysine residues [63,64]. Therefore, applying the concept described in the introduction section (Figure 1) [15], compounds with shorter fatty acid chain (octanoic acid, C8) and additional hydrophobic amino acid residues at the N-terminus were synthesized to obtain more selective and active compounds than those without modification with similar lipophilicity (compounds **B5**, **B6**, **C5**, and **C6**). The adjusted retention times (tR’) of **B6** and **C6** were similar to those of **B2** and **C2** (decanoic acid, C10). The hydrophobicity of analogs with phenylalanine residues (**B5**, **C5**) were higher; however, tR’ values were much closer to those of surfactants with decanoic acid residues than those with dodecanoic acid (Table 1; **B3** and **C3**). The hemolysis (HC_50_) and cytotoxicity (IC_50_) of gemini surfactants with norleucine (**B6** and **C6**) were similar to those of **B2** and **C2**. Lipopeptides with phenylalanine residues (**B5** and **C5**) were noticeably more hemolytic and **C5** was more cytotoxic than **C2** but less cytotoxic than **C3**. Generally, surfactant **B5** showed a reduced antimicrobial activity as compared to that of the corresponding surfactant **B2**, but compound **B6** exhibited a slightly increased activity against *K. pneumoniae* and *P. aeruginosa*. In effect, **B6** has the highest selectivity index (41.04, similar to the SI of **C7**) considering the IC_50_ and MIC of the compound against *P. aeruginosa*. Interestingly, both **C5** and **C6** were distinctly more active against *K. pneumoniae* (MICs 32 and 64 µg/mL, respectively) than **C1**–**C4** lipopeptides (inactive, MIC >256 µg/mL). In our previous study, the antimicrobial activity of gemini lipopeptides with an l-cystine diamide spacer and lysine residues (C12-KKC-NH_2_ and C14-KKC-NH_2_) was evaluated [65]. MICs against planktonic cultures of *S. aureus* ATCC 25923 and *P. aeruginosa* ATCC 9027 were usually higher than those of **C3** and **C4** being analogous surfactants but with arginine residues (except for C14-KKC-NH_2_ against *S. aureus*, MIC = 32 µg/mL vs. 64 µg/mL of **C4**). This shows that arginine residues can be more effective than lysine ones in gemini surfactants used against the planktonic cells of these strains. Elongation of the fatty acid chain (**C12** < **C14** < **C16**) in the series with an l-cystine diamide spacer and the lysine residues resulted in a reduction of the antimicrobial activity [65]. This finding is consistent with those of the present study, since the antimicrobial activity decreased in series **C** with increasing chain length (**C2** < **C3** < **C4**).

**Figure 6:**
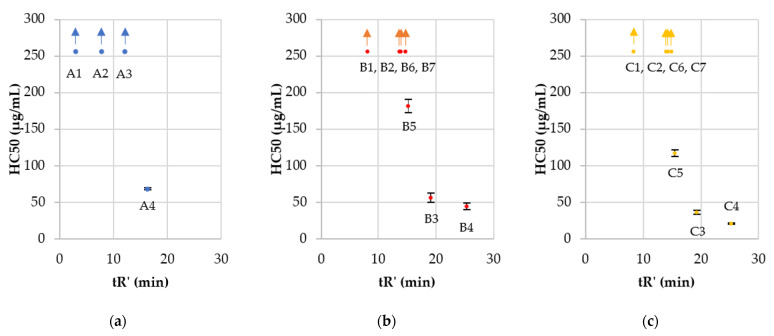
Hemolysis (HC_50_) vs. adjusted retention time (tR’): (**a**) USCL—parent molecules (series **A**); (**b**) gemini lipopeptides with l-lysine amide spacer (series **B**); (**c**) gemini lipopeptides with l-cystine diamide spacer (series **C**). Arrows indicate that HC_50_ was above the applied concentration range.

The majority of the tested surfactants did not exhibit any hemolytic activity in the applied concentration range (HC_50_ >256 µg/mL). In the present study, monomeric lipopeptides appeared to be less hemolytic than the corresponding gemini surfactants (those with identical fatty acid residues) e.g., **A4**, **B4**, and **C4** have HC_50_ values of 68.43 ± 1.41, 44.12 ± 4.70, and 20.89 ± 1.06 µg/mL, respectively. Indeed, in the previous studies on arginine-based surfactants performed by Mitjans et al. (two arginine residues per molecule and a 1,3-diaminepropane spacer), geminis were more hemolytic than the corresponding monomeric ones [66]. The increasing hydrophobicity in each group (Figure 6A–C) resulted in enhanced hemolysis. Similarly, the HC_50_ of Arg-based gemini surfactants studied by Mitjans et al. was increasing with the number of carbon atoms in fatty acid chains reaching the top HC_50_ value of 12.5 ± 0.4 µg/mL (surfactant with dodecanoic acid). Hemolysis seems to be correlated with peptide hydrophobicity as reported in the literature [15,66]. The authors indicate that an essential parameter associated with the hemolysis is the CMC of the surfactant. The CMC of both monomeric and dimeric surfactants is generally related to the fatty acid chain length and hydrophobicity (i.e., hydrophile–lipophile balance) [67,68]. Past studies revealed that the spacer length can affect the size of aggregates that are formed by surfactant molecules and consequently antimicrobial and hemolytic activities. Longer spacers led to an increasing aggregates size and thus reduced interactions with membranes [69]. l-cystine spacers are longer than the l-lysine and geminis ones with l-cystine diamide and are more hydrophobic than the corresponding compounds with the l-lysine amide and thus can affect the aggregate size.

**Figure 7:**
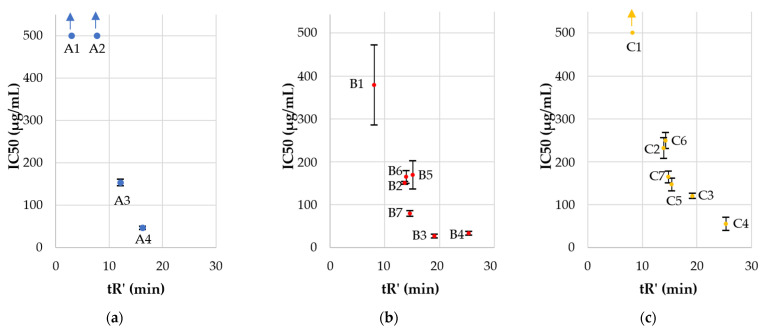
Cytotoxicity (IC_50_) vs. adjusted retention time (tR’): (**a**) USCL—parent molecules (series **A**); (**b**) gemini lipopeptides with l-lysine amide spacer (series **B**); (**c**) gemini lipopeptides with l-cystine diamide spacer (series **C**). Arrows indicate that IC_50_ is above the applied concentration range.

Two USCLs (**A1** and **A2**) were nontoxic to HaCaT cells, but their antimicrobial activity was also above the applied concentration range. Moreover, one of the gemini surfactants (**C1**) showed IC_50_ above 500 µg/mL. As in the hemolysis, IC_50_ decreased in each series with an increasing hydrophobicity of the compound. Gemini surfactants with the l-cystine diamide spacer (**C**) exhibited lower cytotoxicity (IC_50_) than the corresponding surfactants with l-lysine amide spacer (**B**) despite their higher hydrophobicity (Table 2, Figure 7), but no such effect was seen in human red blood cells. The surprisingly low cytotoxicity of gemini surfactants with cystine-based spacer can be explained by the lability of cystine and potential reduction to thiol due to the pH, enzymes, and cell metabolism [70,71]. l-cystine diamide is widely used as a gemini spacer, but it is sensitive to the reducing as well as oxidizing conditions. The reduced lipopeptides would have a general structure, (Fatty acid)-RRC-NH_2_, similar to that of group **A**—monomeric lipopeptides, but the resulting monomeric lipopeptides have an additional cysteine residue at the C-terminus. The lipopeptides of series **A** with comparable hydrophobicity (cLogPs of **A1**, **A2**, **A3**, **A4** are −1.05 ± 0.62, 0.01 ± 0.62, 1.07 ± 0.62 and 2.14 ± 0.62, respectively) to the reduced form of surfactants of group **C** (cLogPs are presented in Table 3). They have relatively low cytotoxicity.

The hydrophobicity is a crucial parameter, and therefore, the lowered IC_50_ of series **C** compounds can be explained by the reduction of gemini surfactants to monomers species similar to compounds of series **A**. It has been shown that cystine can be reduced by the intracellular environment and glutathione. This phenomenon is under study owing to its possible application to create amphiphilic molecules to drug delivery. In view of its instability inside the cell (GSH—reduced glutathione), the disulfide can be degraded to release a drug [72,73]. Hypothetically, surfactants with cystine spacer (series **C**) can be candidates for further studies as drug carriers owing to their ability to form micelles. Moreover, cationic antimicrobial peptides have been shown to promote the generation of reactive oxygen species (ROS) that can induce extracellular export of GSH [74,75]. Studies on the UVA effect on HaCaT have shown that GSH efflux itself is involved in HaCaT membrane rearrangement (phosphatidylserine exposition) and an increased plasma membrane permeabilization [76]. Moreover, a fraction of the damaged cells can release GSH into the environment to inactivate surfactants with l-cystine diamide spacer through a reductively split in reducing conditions. Undoubtedly, the stability of surfactants is another essential issue that should be considered. In this study, the serum stability and antimicrobial activity of lipopeptides in the presence of human serum were tested to learn how its enzymatic activity and possible peptide–protein binding would affect biological properties.

**Figure 8:**
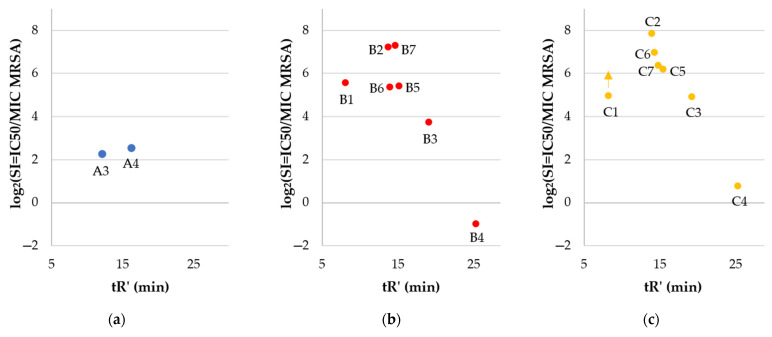
The log_2_ of selectivity indexes of surfactants vs. adjusted retention time (tR’): Cytotoxicity (IC_50_) vs. adjusted retention time (tR’): (**a**) USCL—parent molecules (series **A**); (**b**) gemini lipopeptides with l-lysine amide spacer (series **B**); (**c**) gemini lipopeptides with l-cystine diamide spacer (series **C**). The selectivity included IC_50_ HaCaT and MIC of compounds against MRSA (Methicillin-resistant *Staphylococcus aureus*, ATCC 33591). Arrows indicate that IC_50_ is above the applied concentration range.

The selectivity of compounds to pathogens over human keratinocytes was estimated (Table 2, Figure 8). Selectivity indexes (SIs) were calculated as IC_50_, HaCaT/MIC (SI HaCaT), or HC_50_/MIC (SI hRBCs).

The majority of gemini surfactants (except **B4** and **C4**) were more selective than monomers (series **A**). Once again, hydrophobicity turned out to be a key parameter for biological properties. Compounds with a higher retention time (tR’) have diminished selectivity indexes than those more hydrophilic ones. The antimicrobial activity of **B4** and **C4** is reduced as compared to that of gemini surfactants with shorter fatty acid chains due to the self-assembly and formation of aggregates that can reduce antimicrobial activity. On the other hand, those peptides exhibited high cytotoxicity, which made them the least selective. It is noteworthy that in vitro studies cannot be expected to give a final statement on the toxicity of compounds. Nevertheless, some attempts are made to predict irritant potential with in vitro methods to select compounds that can be used in pharmaceutical and cosmetic formulations [77]. In this article, cytotoxicity to HaCaT cell line and hemolysis were determined to give the first evaluation of compounds to select the most promising surfactants for further in vivo studies. It can be stated that the most potent and selective dimers are those with the highest selectivity indexes, which were determined for **B2**, **B7**, **C2**, and **C6** (150.03, 157.56, 231.17, and 124.42, respectively). Arginine-based gemini surfactants tend to be more biocompatible, biodegradable, and less toxic to aquatic organisms than structurally similar geminis of quaternary ammonium salts [62,78]. Antimicrobial peptides are usually considered to be used for topical treatment due to their short half-life (degradation by proteases) [79]. Undoubtedly, cationic detergents may cause irritant skin responses [80]. Polymyxin B as a cationic surface-active antibiotic is already used in clinical practice as an ingredient of topical ointment to treat skin infections. Studies on polymyxin B support the thesis that this antibiotic is safe to use in this particular application [81]. Toxic effects and side effects can be modulated with concentration (dose) and formulation and depend on the route of administration, exposure time, and type of cells that are exposed to the compound. The observed toxicity of polymyxins is different in topical application and an intramuscular injection [82]. Analyses on interactions between lipid monolayers showed that arginine-based gemini surfactants have higher penetration capacity into DPPG (1,2-dipalmitoyl-sn-glycero-3-[phospho-rac-(1-glycerol)] sodium salt) and lower into DPPC (1,2-dipalmitoylsn-glycero-3-phosphocholine) than monomeric arginine-based surfactant. Anionic lipids (DPPG) are characteristic for bacterial membranes and zwitterionic (DPPC) for mammalian ones. These results indicate the critical importance of molecule charge (electrostatic forces) and partially explain different antimicrobial activities and cytotoxicity between monomers and dimers [61]. It was shown that cationic antimicrobial lipopeptides can be effective in the treatment of wound bacterial infection without adverse effects on porcine skin [83]. Recent studies on antimicrobial arginine-based gemini surfactants indicate an interesting possibility to incorporate them into vesicles together with anionic amphiphiles (catanionic vesicles), resulting in improved selectivity [84]. On the other hand, cationic micelles and liposomes injected intravenously into rats result in DNA damage in lungs and spleen [85]. It is well known that guanidine groups of arginine and gemini surfactants can effectively interact with DNA and are used in gene transfection [86,87]. Considering those facts, compounds used in this study should be examined as potentially genotoxic. Another aspect that can be examined with arginine-rich compounds is their potential induction of inflammatory responses in human whole blood, including tumor necrosis factor-alpha, through LPS-induced monocyte activation [88].

### 2.3. Antimicrobial Activity in the Presence of Serum

Minimal inhibitory concentrations of lipopeptides were determined in the presence of human serum to evaluate the effect of enzymatic degradation and protein binding of lipopeptides on antimicrobial activity. Serum concentrations were 1% and 10% (*v*/*v*). *S. aureus* (ATCC 33591), *P. aeruginosa* (ATCC 9027), and *C. glabrata* (ATCC 15126) were selected to this study. The results are presented in Table 4.

**Figure 9:**
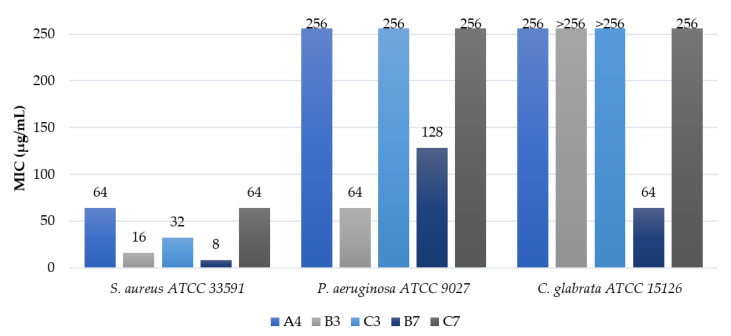
Minimum inhibitory concentrations (MICs) of selected compounds in the 10% (*v*/*v*) normal human serum.

Generally, it can be stated that the increasing serum concentration results in suppressing antimicrobial activity. Some lipopeptides were inactive (MICs >256 µg/mL) in the medium supplemented with the 10% normal human serum. It is noteworthy that even the 1% (*v*/*v*) serum supplementation usually caused a noticeable increase in MIC. This effect was previously reported for antimicrobial peptides (e.g., human β-defensin) and lipopeptides (e.g., daptomycin) [89,90]. This phenomenon is explained as a result of some processes, such as the binding of serum protein to pathogen’s cell surface (protection form antimicrobial agents), or to AMPs, which reduce the free molecules able to disrupt pathogens membrane, or degradation of the peptides by serum proteases [91]. Several studies were devoted to the effect of medium supplementation with serum albumins (bovine serum albumin (BSA), human serum albumin (HSA)) on antimicrobial activity of short cationic peptides and lipopeptides, which usually led to their reduction [91,92,93]. It was demonstrated that HSA was characterized by 11 binding sites for medium-chain fatty acids (C10-C14) [94]. Interestingly, for **B4** and **C4** (gemini surfactants with tetradecanoic acid residues), supplementation of the medium with 1% (*v*/*v*) human serum potentiated their antimicrobial activity against *S. aureus* ATCC 33591; however, no such effect was noticed for other microorganisms tested. Similar tendencies were reported by Richie et al. for amphotericin B against *C. albicans* ATCC 24433 in RPMI-1640 medium supplemented with 1% (*v*/*v*) human serum [95]. However, a reduced activity of **B4** and **C4** was found at higher serum concentration (10%, *v*/*v*). Ghobrial et al., in their studies on lantibiotic MU1140, demonstrated that the potentiation of antimicrobial activity in the presence of serum also varied between strains [96]. Compound **A4** is the most active linear USCLs (series **A**), but its MICs in 10% (*v*/*v*) serum are relatively high (64 and 256 µg/mL). Supplementation with serum resulted in a three- or four-fold higher MICs. Some of the gemini surfactants (**B2–B4**, **C3**, and **C4**) exhibited moderate antimicrobial activity (16–64 µg/mL) against *S. aureus* in the presence of serum. Again, a gemini surfactant with branched fatty acid chains and an l-lysine amide spacer (**B7**) was the most active compound against *S. aureus* in 10% (*v*/*v*) serum with an MIC of 8 µg/mL. The antimicrobial activity of the **C7** analog with an l-cystine diamide spacer in identical conditions was distinctly lower (MIC = 64 µg/mL). Similarly, MICs of **B2**–**B4** in a medium supplemented with serum were lower than those of the corresponding analogs of series **C** (**C2**–**C4**). It can be concluded that *P. aeruginosa* and *C. glabrata* were substantially more resistant than *S. aureus*. Serum supplementation strongly inhibited antimicrobial activity against these two strains. A few compounds showed a moderate-to-low antimicrobial activity at 10% (*v*/*v*) serum, and only two of them (**B3** and **B7**) inhibited growth at concentrations below 256 µg/mL. The most active compounds were selected from each group, and their MICs in the presence of 10% serum are displayed in Figure 9 to facilitate the comparison of antimicrobial potency between three series (**A**, **B**, **C**).

In general, gemini surfactants were found to be more active than monomeric lipopeptides in the medium, either with or without human serum supplementation. Moreover, gemini surfactants with l-lysine amide spacers seem to be more effective than the corresponding surfactants with l-cystine diamide spacers in the serum-supplemented medium. Lipopeptide **B7** appeared to be the most potent compound owing to its outstanding antimicrobial activity against *S. aureus* and *C. glabrata*, which was determined in the serum-enriched culture medium. The only surfactant that exceeded the antimicrobial activity of **B7** against *P. aeruginosa* was compound **B3** (Table 4, Figure 9). Presumably, **B7** with branched fatty acid residues does not interact with HSA binding sites as effectively as do surfactants with straight-chain fatty acids.

### 2.4. Antibiofilm Activity

Biofilm is associated with life-threating infections due to its outstanding resistance to antibiotics. In this study, the antibiofilm characteristics of USCLs and gemini surfactants were evaluated. The results expressed as minimum biofilm eradication concentrations (MBECs) are shown in Table 5.

Lipopeptide **A4** was the most effective USCL in series **A,** this being consistent with the tendencies observed in MIC assay. The biofilm of Gram-positive strains was the most susceptible to lipopeptides. In general, the most efficient compounds against the biofilm of Gram-positive bacteria were **A4**, **B2**, **B5**–**7**, **C2**, and **C5**–**7**. The biofilm of *S. aureus* ATCC 33591 (MRSA) was more sensitive to the vast majority of compounds than that of *S. aureus* ATCC 25923. In our previous study on the antimicrobial activity of N-terminally lipidated analogs of cationic peptide KR-12, the biofilm of the MRSA (ATCC 33591) strain was more susceptible to lipopeptides than that of MSSA (ATCC 25923) [97]. This can be the result of a greater thickness of *S. aureus* ATCC 25923 biofilm than that of *S. aureus* ATCC 33591. Moreover, their susceptibility depends on the biofilm maturity and the antibiotic used [98]. It was found that the antibiofilm activity of antibiotics (e.g., daptomycin, gentamicin) can be potentiated (synergistic effect) through supplementation with cationic antimicrobial peptides and lipopeptides [99,100]. Therefore, further studies should be extended to examine antibiofilm activity of gemini surfactants combined with antibiotics. The biofilm of *E. faecium* ATCC 700221 (vancomycin resistant—VRE strain) exhibited similar susceptibility to lipopeptides to the MRSA strain. Studies on bacterial biofilm showed that the mass of the VRE biofilm cultured in MHB was considerably lower than that of the *S. aureus* stains [101]. This can explain the relatively high sensitivity of VRE biofilm to lipopeptides. In our previous study, gemini surfactants with l-cystine diamide spacers and lysine residues (C12-KKC-NH_2_ and C14-KKC-NH_2_) revealed a slightly higher antibiofilm activity (MBEC) against *S. aureus* ATCC 25923 and *P. aeruginosa* ATCC 9027 than that of the corresponding surfactants with arginine residues **C3** and **C4** (except for C12-KKC-NH_2_ and **C3**, MBECs against *P. aeruginosa* were >512 and 512, respectively) [65]. In general, the biofilm of *P. aeruginosa* was the most resistant to lipopeptides (MBEC ≥128 µg/mL). The *P. aeruginosa* biofilm was extraordinarily resistant to cationic lipopeptides owing to its structure and composition. For example, Di Domenico et al. reported that *P. aeruginosa* ATCC 9027 was a high biofilm producer, while *K. pneumoniae* ATCC 700603 was moderate. This could be a key factor of such tendency [102]. Resistance to antimicrobial peptides and lipopeptides can be the result of biofilm matrix components (eDNA) that successfully inhibit the activity and penetration of cationic antibiotics [103]. Moreover, *P. aeruginosa* ATCC 9027 was a mono-rhamnolipid and alginate producer [104,105]. The lack of *mucA* gene affected the constitutive expression of alginate biosynthetic genes [106]. One of the explanations provided is that the negatively charged entities of the biofilm matrix attract positively charged antibiotic molecules to protect bacterial cells [107]. Billings et al. discovered that Psl (D-mannose, D-glucose, and L-rhamnose) as extracellular matrix components of biofilm was partially responsible for resistance to antibiotics including cationic lipopeptides (colistin, polymyxin B) [108]. Psl is responsible for cell–cell and cell–surface interactions as well as the biofilm structure [109]. Biofilms of the remaining Gram-negative strains, *A. baumannii*, *K. aerogenes*, and *K. pneumoniae*, exhibited similar susceptibility to lipopeptides, and **B7** was the most effective compound, while the analog with an l-cystine diamide spacer (**C7**) was markedly less active. The MBEC of **B7** was 128 µg/mL against *K. pneumoniae* and 64 µg/mL against *A. baumannii* and *K. aerogenes.* The remaining compounds were less effective or even inactive. *A. baumannii* and *K. pneumoniae* were classified by Swedan et al. as strong biofilm producers, this offering an explanation of their relatively low susceptibility [110]. Persister cells are believed to be one of the factors that can contribute to high biofilm resistance to antibiotics. Those cells suppressed the metabolism and are dormant variants of regular cells that form stochastically in microbial populations and are highly tolerant to antibiotics [111,112]. Michiels et al. found that the antibiotic treatment of biofilms of ESKAPE strains, including *S. aureus* ATCC 33591 and *K. aerogenes* ATCC 13048, increased levels of persister cells [113]. In effect, biofilms with those cell variants can effectively be renewed when antibiotic is removed. Fungal strains such as *C. albicans* and *C. glabrata* are associated with serious infections, especially in immunocompromised patients. Both strains can form biofilm, for example on medical devices, catheters, and prosthetic joints, but also on tissues involved in the development of vaginal and oral candidiasis [114]. Biofilm development complicates therapy and makes antibiotics significantly less effective. The main reason of *Candida* sp. biofilm resistance is the extracellular matrix (ECM), which sequesters antifungal drugs. ECM is composed of carbohydrates, glucose, β-1,3-glucan, extracellular DNA, proteins, hexosamine, and uronic acid [115,116,117]. Other mechanisms of biofilm resistance can be associated with e.g., the upregulation of drug efflux pumps and persister cells [118]. Moreover, biofilms of *C. albicans* involve blastospores, hyphae, and pseudohyphae, while the biofilm of *C. glabrata* contains exclusively blastospores [119]. In this study, the biofilm of *C. glabrata* turned out to be more resistant to lipopeptides than that of *C. albicans*, whereas the antimicrobial activity against planktonic cultures was similar between both strains. In our previous studies, MBEC of USCLs against biofilms were almost identical between these two strains [120]. It was noticed that amphotericin B, which disrupts the plasma membrane, preferentially affects germ tubes [121]. Moreover, Suci and Tyler found that membrane-active chlorhexidine penetrates filamentous form more rapidly than blastospores [122]. This is why chlorhexidine has a positive charge from guanidine groups (they also occur in the arginine side chain), the hydrocarbon chain (6 carbon atoms) connecting guanidine, and two opposite 4-chlorophenyl entities as hydrophobic fragments of the molecule. This architecture is similar to those of gemini surfactants. Hypothetically, the compounds used in this study interact differently with blastospores and hyphae (pseudohyphae), and therefore, their antibiofilm activity against filamentous (*C. albicans*) and non-filamentous fungi (*C. glabrata*) is different. Compounds **A4**, **B3**–**5**, **C3**, and **C4** are most effective against yeast biofilms. Interestingly, **B5** with phenylalanine residues is substantially more active against *C. albicans* biofilm than **B6** (analog with norleucine), while the antibiofilm activity of these compounds was comparable for the remaining strains. Moreover, it can be deduced that an effective eradication of *Candida* strains biofilm requires compounds with longer fatty acid chains than those effective against bacterial biofilms. The lowest MBECs of surfactants against *Candida* spp. was noticed for compounds with dodecanoic and tetradecanoic acid residues (**B3**, **B4**, **C3**, **C4**), while against bacterial biofilm, the most active compounds consist of decanoic acid residues (**B2** and **C2**). This observation is also consistent with the results obtained for planktonic cultures of microorganisms. Similarly to planktonic cultures, gemini surfactants with branched fatty acid chains were less active against yeast biofilm (64–256 µg/mL) than compounds with straight fatty acid chains (16–128 µg/mL).

### 2.5. Membrane Permeabilization

**Figure 10:**
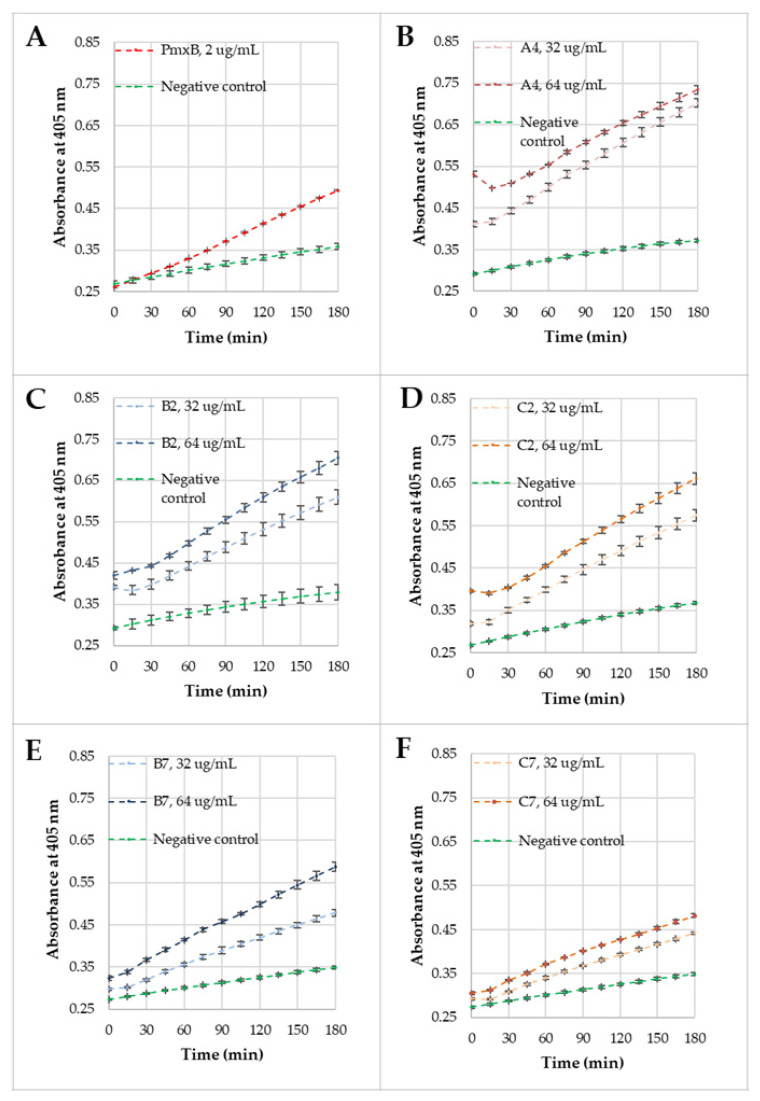
Outer membrane (OM) permeabilization kinetics. (**A**) Polymyxin B at 2 μg/mL; (**B**) **A4** at 32 and 64 μg/mL; (**C**) **B2** at 32 and 64 μg/mL; (**D**) **C2** at 32 and 64 μg/mL; (**E**) **B7** at 32 and 64 μg/mL; (**F**) **C7** at 32 and 64 μg/mL.

**Figure 11:**
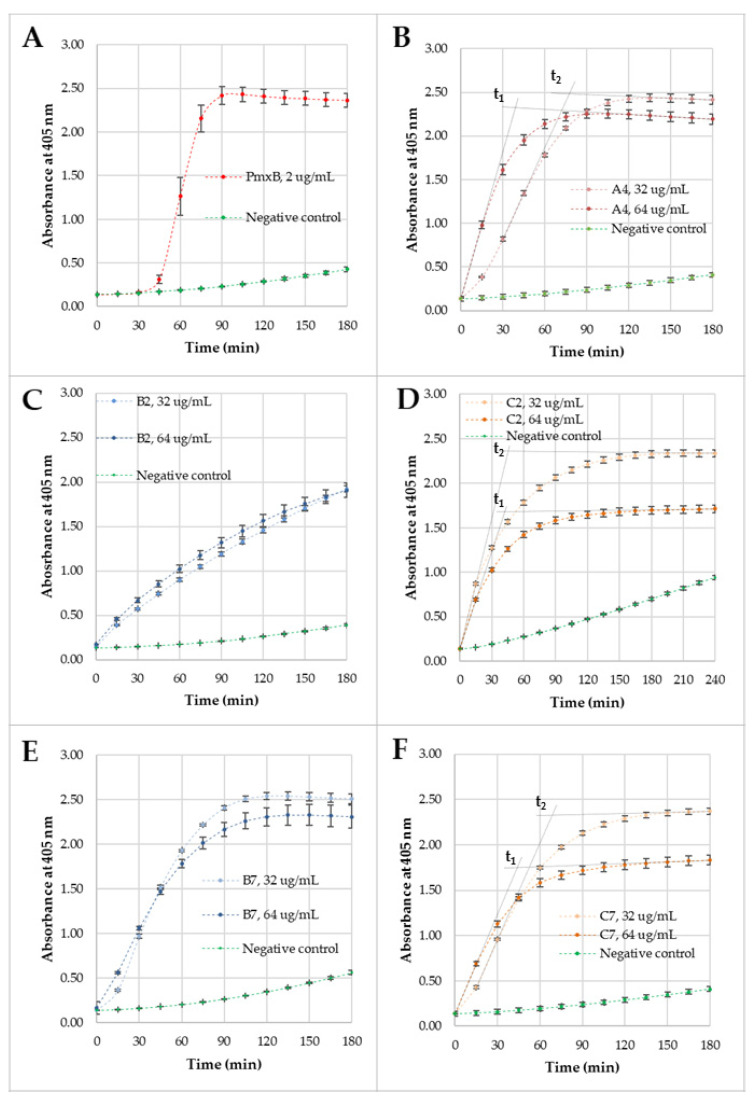
Inner membrane (IM) permeabilization kinetics. (**A**) Polymyxin B at 2 μg/mL; (**B**) **A4** at 32 and 64 μg/mL; (**C**) **B2** at 32 and 64 μg/mL; (**D**) **C2** at 32 and 64 μg/mL; (**E**) **B7** at 32 and 64 μg/mL; (**F**) **C7** at 32 and 64 μg/mL.

The permeabilization of *E. coli* ML-35 (ATCC 43827) membranes was studied for selected compounds (**A4**, **B2**, **C2**, **B7**, and **C7**) to compare their activity and membrane disruption over time. Lipopeptide **A4** appeared to be the most active among the monoacylated USCLs, while **B2**, **C2** and **B7**, **C7** were the most effective antimicrobial gemini surfactants with straight and branched fatty acid chains, respectively. Moreover, each gemini surfactant with the lysine-based spacer had a corresponding analog with a l-cystine diamide spacer in series **C**. The MICs against the *E. coli* strain for these compounds ranged between 32 and 64 µg/mL (**A4**, **B2**, **C2**, **B7**—32 µg/mL, and **C7**—64 µg/mL) and therefore, outer (OM) and inner membrane (IM) permeabilization studies were performed at these two concentrations. For OM and IM permeabilization, CENTA and ONPG were used, respectively. The permeabilization of OM causes the release of periplasmic β-lactamase, which hydrolyzes CENTA’s β-lactam ring to form a chromophore, 2-nitro-5-thiobenzoate (TNB) anion, while the disruption of IM results in the release of β-galactosidase, which hydrolyzes ONPG into galactose and a chromophore, ortho-nitrophenol (ONP). Both products of enzymatic reaction absorb light at 405 nm. A phosphate buffer saline (PBS) solution with ONPG/CENTA was used as a negative control, and polymyxin B (PmxB) at MIC (2 µg/mL) was used as a positive control. The results of OM and IM permeabilization experiments are shown in Figure 10 and Figure 11, respectively.

OM permeabilization experiments were performed over 3 h. It can be noticed that absorbance at 405 nm for negative control increased by 0.1 AU. Polymyxin B as a positive control gave a significantly higher increase than the negative control (ca. 0.25 AU). Absorbances at t_0_ for positive and negative control were comparable. The permeabilization of OM was analyzed at two concentrations of compounds. It was found that increasing concentration caused enhanced membrane disruption due to a higher absorbance at corresponding time intervals and a greater slope of the curve at a 64 µg/mL concentration. Interestingly, there are differences at t_0_ between compounds and the same surfactants at different concentrations. Measurements started immediately after the cells were mixed with lipopeptides or geminis and a substrate (CENTA) in the buffer. Moreover, surfactants did not absorb at 405 nm (fatty acid residues and amino acids). A disulfide bond can absorb near 250 nm, but it is still far below the wavelength that was used in this experiment [123]. Presumably, these differences result from the influence of surfactants on the UV absorption of a CENTA hydrolysis product (2-nitro-5-thiobenzoate). In Figure 10B–D, after 15 min of incubation, there appears a distinct minimum on the plot. Compounds at these concentrations do not form micelles (Table 2) but are still engaged in other processes such as interactions with bacterial membrane and other cell elements (fraction F1) and interactions with the dye and solvent (CENTA before and after hydrolysis; fraction F2). Hypothetically, the decrease in the 405-nm absorbance after 15 min of incubation results from a reduction of fraction F2 that, as we presumed, can have an influence on the dye and elevate light absorption at 405 nm (direct interactions with TNB anion, e.g., ion-ion, or effect on pH). A further increase (after 15 min) of absorption is evidently related to membrane disruption and activity of the released β-lactamase. Surfactants **B2** and **C2** (Figure 10C,D) caused a similar effect on OM at both concentrations, but surfactant **B7** disrupts the membrane more efficiently than **C7** at the same concentrations (Figure 10E,F). Generally, all compounds disrupt the outer membrane either immediately or after 15 min. This can be deduced from the linear increase in the 405-nm absorbance just after 15 min of incubation. First of all, curves of compounds significantly differ from those of negative control and are similar to that of the positive control (PmxB). The concentration of substrate decreases in time due to an enzymatic reaction that manifests itself as an increase in the 405-nm absorbance. There is no plateau in Figure 10, and therefore, CENTA is not completely hydrolyzed in any case. Hypothetically, the linear increase in absorbance results from the complete release of enzyme ([E]) and high concentration of substrate ([S]) over incubation time. Moreover, the curves in Figure 10 (considered for individual compounds) are almost parallel, this providing another evidence in favor of the hypothesis. The slightly different slopes can be interpreted in terms of different cell concentrations between wells/samples and thus different maximum enzyme concentration. Moreover, it seems that compounds can interact with the enzyme and/or product (TNB) influencing the activity and/or effectiveness of light absorption e.g., through changing the pH or interacting with ionized groups or hydrophobic fragments of those molecules, although this remains a matter for further discussion. It was found that the curves represent zero-order reaction kinetics ([E]>>[S]), especially after the first 15 min. Similar results were obtained for USCLs consisting of palmitic acid and cationic amino acid residues (lysine and arginine) [120] and other cationic antimicrobial peptides (e.g., NCP-3, ADP) [124,125].

All compounds at their MICs caused IM perturbation in shorter time than that of PmxB. The positive control showed a rapid increase in absorbance at 405 nm after 30 min of incubation, while all the remaining compounds disrupted IM immediately. Furthermore, maximum absorbance was reached after 90–120 min of incubation (except **B2**). It is concluded that **A4** and gemini surfactants pass easily across the OM and periplasm, but PmxB needs more time to reach the IM. Interestingly, the maximum absorbance for **C2** differs significantly between two concentrations. Incubation of the *E. coli* strain with ONPG and **C2** at 32 µg/mL resulted in a higher absorbance of the ONP at each time interval than that of **C2** at 64 µg/mL, this showing that raising the concentration of **C2** reduced the membrane damage or the reaction kinetics. These findings cannot be interpreted as a result of differences in ONPG or bacteria concentration, because both were prepared in the same experiment in one stock for both surfactant concentrations. To evaluate **C2**, IM permeabilization kinetics, the 405-nm absorbance was recorded over an additional hour to reach plateau. However, the discrepancies in the absorbance peaks can be misleading, as they cannot result from different permeabilization kinetics, since maximum absorbances are only limited by the substrate (ONPG) concentration, which was identical for each experiment. Additional experiments on **C2** IM permeabilization were performed at different concentrations (128 and 256 µg/mL) over 4 h (Supplementary Materials, Figure S7). Evidently, the higher the **C2** concentration was, the lower the 405-nm absorbance became. Moreover, we found out that maximum absorbance was linearly related to the binary logarithm of concentration (R^2^ = 0.9676; Supplementary Materials, Figure S8). These results support the thesis that surfactants can affect chromophores by changing their photo-physical properties. Presumably, this is the result of a variable environment as reported in the literature [126]. The ONP displays different UV spectra in variable pH, and thus, it can be deduced that compounds (being salt of trifluoroacetic acid and arginine-rich surfactant) affect the degree of ONP ionization. To overcome this issue, we suggest determining incubation time (t_i_) corresponding to the intersection of two tangents. The first tangent passes through t_0_ (the slope of this tangent is associated with membrane disruption and released enzyme), while the other is based on the last two measurements (tangent to the plateau; this tangent is associated with the amount of product). The determined t_i_ values are unaffected by the surfactant influence on the ONP ionization state because t_i_ remains stable even when molar fractions of protonated/deprotonated ONP are varying (ONP/ONP^-^). If the compounds had an indistinguishable IM kinetics, then the determined t_i_ would have been identical. On the other hand, a lower t_i_ indicates a faster kinetics and, respectively, a higher t_i_ (Supplementary Materials, Figure S9). Two tangents to each curve are shown in Figure 11D. The intersection of those tangents allows determining the time (t_1_, t_2_) that seemingly can be used to compare those two reactions. In fact, t_1_ and t_2_ are comparable (42.0 min and 45.7 min, respectively), and thus, it seems that **C2** has congruent membrane permeabilization kinetics at both concentrations. Again, there is a linear correlation between t_i_ at different **C2** concentrations and the binary logarithm of concentration (R^2^ = 0.9622; Supplementary Materials, Figure S10). These results indicate that raising the concentration of **C2** results in elevated IM kinetics even if the 405-nm absorbance falls. Similar differences were noticed for **C7**. Maximum absorptions at 405 nm are much lower for **C7** at 64 µg/mL than that recorded at 32 µg/mL. Again, t_1_ is lower than t_2_ (43.9 min vs. 68.6 min), but this time is much longer than that for **C2**. IM permeabilization kinetics is more pronounced at MIC of **C2** (64 µg/mL) than at ½ MIC. Similarly, **A4** (Figure 11B) showed a higher IM permeabilization kinetics at 64 µg/mL than that of 32 µg/mL (t_1_ vs. t_2_, 39.0 min and 77.6 min, respectively). In general, all surfactants permeabilize both IM and OM and exhibited comparable lithic action on membranes. Similar results were obtained in experiments with USCLs and other antimicrobial cationic peptides, but raising the peptide concentrations did not always lead to a more pronounced inner membrane disruption and can differ significantly between monomeric and dimerized antimicrobial peptides [120,127]. Presumably, this can be due to some specific interactions with enzymes, substrates, or reaction products. Undoubtedly, these results support the thesis that USCLs and gemini surfactants are potent IM and OM permeabilizing agents.

### 2.6. Serum Stability

**Figure 12:**
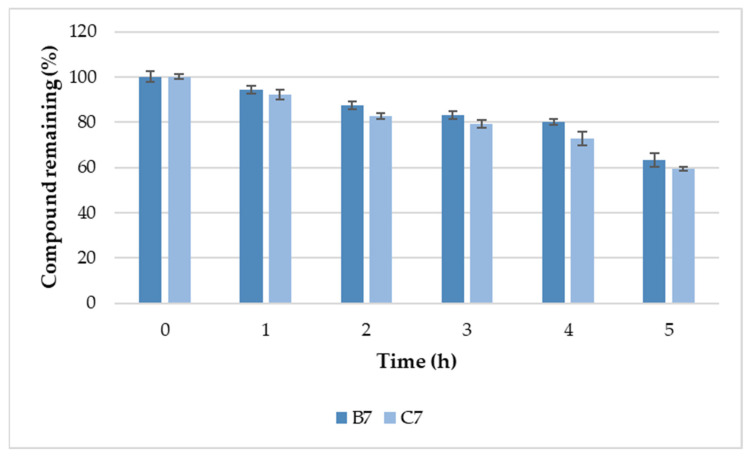
Minimum inhibitory concentrations (MICs) of selected componds in 10% (*v*/*v*) normal human serum.

Compounds **B7** and **C7** were selected for further serum stability studies owing to their high antimicrobial activity (Table 2, Table 4, Table 5 and Figure 9), and relatively high selectivity indexes (Table 2, Figure 8). In fact, these compounds are analogous surfactants originating from two different series (**B7** with l-lysine amide and **C7** with l-cystine diamde). The results of analysis of **B7** and **C7** stability in human serum are presented in Figure 12.

Serum stabilities of both gemini surfactants (**B7** and **C7**) were almost identical (specific values are attached as Supplementary Materials, Table S2). After 5 h of incubation with serum, the amounts of remaining compounds were 63.2 ± 2.9% and 59.4 ± 1.0% of initial concentrations of **B7** and **C7**, respectively. It can be speculated that the remaining gemini surfactants with l-lysine amide spacers exhibited serum stability comparable to that of surfactants with l-cystine diamide spacers. Dimerization through disulfide bridges and the disulfide cyclization of linear peptides have been known to improve peptide stability to proteolytic degradation [128,129]. Indeed, our results support the thesis that the disulfide can be relatively resistant to serum proteolytic degradation. Moreover, a relatively high resistance of the surfactant to proteolysis can be explained as an effect of the acylation of N-terminal amine groups with fatty acids [130,131].

### 2.7. Self-Assembly Tendency via Molecular Dynamic Simulations

**Figure 13:**
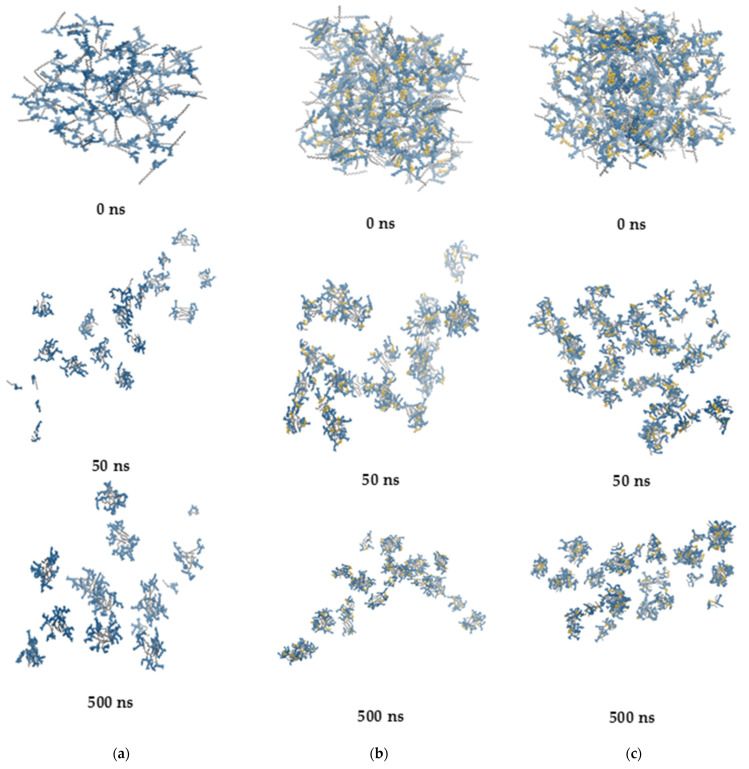
Snapshots from the self-assembly simulations. (**a**) **A3**, (**b**) **B3**, and (**c**) **B7**. Fatty acid tails are in gray, arginines are in blue, whereas the lysine linker is in yellow.

Lipopeptides tend to self-assemble already in the early stages of MD simulations. After ca. 50 ns, the number of clusters drops to a level that is more or less maintained until the end of the MD simulations (Figure 13).

**Figure 14:**
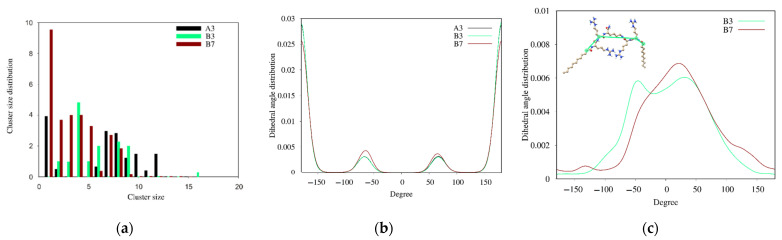
(**a**) Cluster size distribution over the last 50 ns of MD simulations. (**b**) Dihedral angle distribution for all C-C bonds of the hydrocarbon fatty acid tails. (**c**) Dihedral angle distribution of the dihedral angle between the carbon atom of the first methylene group of the N-terminal fatty acid, the alpha carbon of the N-terminal arginine, the alpha carbon of the C-terminal arginine, and the carbon atom of the first methylene group of the C-terminal fatty acid (C2^N-terminal acyl chain^—Cα^N-terminal Arg^—Cα ^C-terminal Arg^—C2 ^C-terminal acyl chain^).

All the lipopeptides studied self-assemble into oligomers and small micelles with an average aggregation number of 6–8 (Figure 14a).

However, **B7** lipopeptide monomers constitute a large part of the entire population. This phenomenon shows that the steric hindrance resulting from branching of the hydrocarbon chain reduces the tendency of **B7** to self-assembly.

In the hydrocarbon chain of fatty acids, three interconverting conformational conformers exist for four consecutive carbon atoms, i.e., one *trans* and two *gauche* conformations. As seen in Figure 14B, the distribution of C-C-C-C dihedral angles is similar for all the lipopeptides. The observed maxima correspond to *trans* (180°), *gauche*- (−60°), and *gauche*+ (60°) conformations. By integrating the dihedral angle distribution, the hydrocarbon chain of dodecanoic acid in both **A3** and **B3** compounds is found to be approximately 81% *trans* and 19% *gauche*, with an even split between the *gauche*- and *gauche*+ conformations. The symmetry of the distribution reflects the achirality of the hydrocarbon chain by analogy to butane [132]. In turn, branching of the hydrocarbon chain in the **B7** lipopeptide leads to a slight increase in the *gauche* fraction up to ca. 24% and an asymmetry between the two *gauche* populations (13% and 11% for *gauche*- and *gauche*+, respectively).

A relative orientation of both acyl chains around the spacer (*cis* and *trans*) in the gemini surfactants is likely to determine a unique self-assembly behavior. Small micelles formed at lower concentration can be cross-linked together by *trans* configuration of the acyl chains into network aggregates with increasing concentrations of the surfactant. However, the gemini surfactant with a long rigid spacer is likely to display opposite abnormal effects. With an increase in surfactant concentration, the *trans* configuration of alkyl tails can transform into the *cis* one and cause the network aggregates to disappear and transform into vesicles or micelles [133,134]. With lipopeptides **B3** and **B7**, a linear lysine-based spacer is not a typical rigid representative. However, as suggested previously, the rigidity of the linear spacer depends on the length of the spacer [135]. Nevertheless, none of the aforementioned phenomena occur with the gemini lipopeptides **B3** and **B7** during the entire simulations, and the acyl chains adopt predominantly *cis* orientation around the spacer (Figure 14C).

### 2.8. Surfactant—Membrane Interactions via Molecular Dynamic Simulations

Three compounds, **A3**, **B3**, and **B7**, were selected for the study of interactions with a Gram-positive bacterial membrane (3:1 1-palmitoyl-2-oleoyl-sn-glycero-3-phosphoglycerol. (POPG): 1-palmitoyl-2-oleoyl-sn-glycero-3-phosphoethanolamine (POPE)). Our purpose was to verify whether or not the connection of two units of analog **A3** by a linker (**B3**) and additionally replacement of the linear fatty acyl chain by a branched chain still containing twelve carbon atoms (**B7**) affected the interactions and mechanism of action.

**Figure 15:**
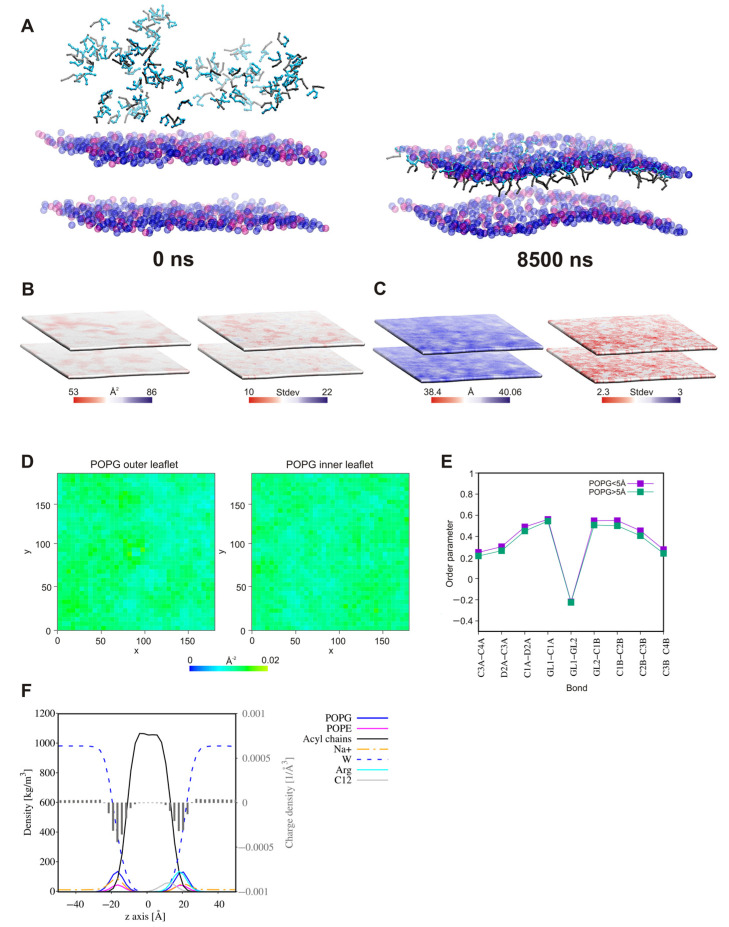
(**A**) Initial and final snapshots from the 3:1 POPG:POPE binding simulations for **A3**. Fatty acid tails are in gray, while arginines are in cyan. Lipid tails were omitted for clarity, and only the POPG and POPE phosphate beads are shown in blue and pink, respectively. (**B**) Local area per lipid (APL) and standard deviations of the local membrane APL and (**C**) local thickness of the bilayer. (**D**) 2D density map of the POPG lipids in the outer and inner leaflets of the POPG:POPE membrane (a grid spacing was set to 5 Å). APL, membrane thickness, and 2D density maps were calculated for phosphate beads of the lipid headgroups. (**E**) Lipid acyl chain order parameters of POPG molecules, separately for the lipids at a distance less than and greater than 5 Å from the lipopeptide molecules. (**F**) Partial density and charge density profiles. All the analyses were performed over the last 100 ns of CG MD simulations. POPE: 1-palmitoyl-2-oleoyl-sn-glycero-3-phosphoethanolamine, POPG: 1-palmitoyl-2-oleoyl-sn-glycero-3-phosphoglycerol.

**Figure 16:**
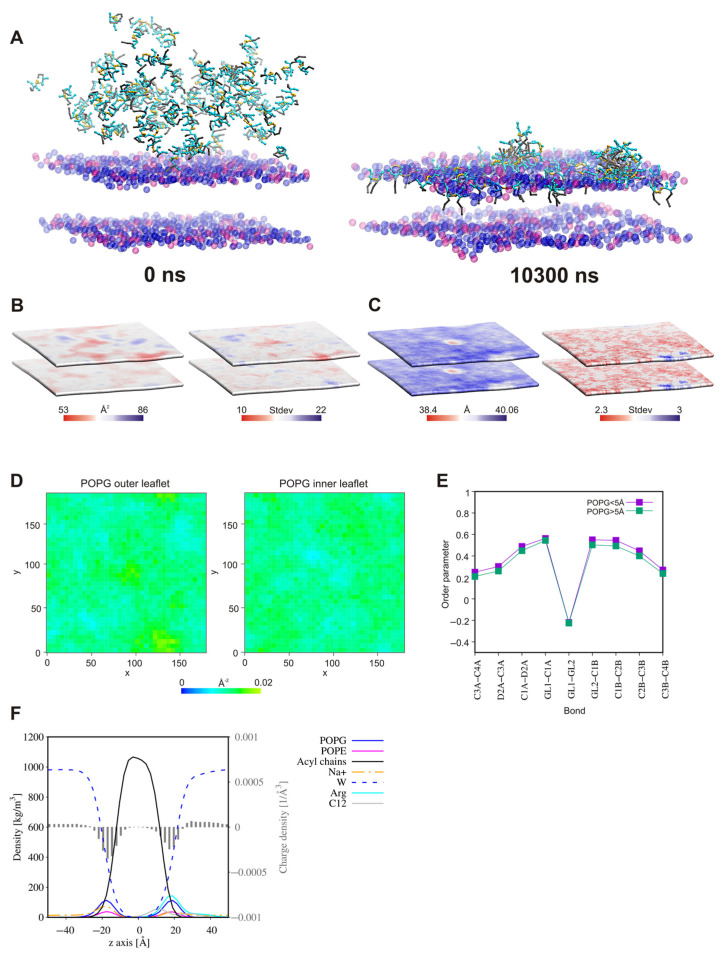
(**A**) Initial and final snapshots from the 3:1 POPG:POPE binding simulations for **B3**. Fatty acid tails are in gray, while arginines are in cyan. Lysine spacers are in orange. Lipid tails were omitted for clarity and only the POPG and POPE phosphate beads are shown in blue and pink, respectively. (**B**) Local area per lipid (APL) and standard deviations of the local membrane APL and (**C**) local thickness of the bilayer. (**D**) 2D density map of the POPG lipids in the outer and inner leaflets of the POPG:POPE membrane (a grid spacing was set to 5 Å). APL, membrane thickness, and 2D density maps were calculated for phosphate beads of the lipid headgroups. (**E**) Lipid acyl chain order parameters of POPG molecules, separately for the lipids at a distance less than and greater than 5 Å from the lipopeptide molecules. (**F**) Partial density and charge density profiles. All the analyses were performed over the last 100 ns of CG MD simulations.

**Figure 17:**
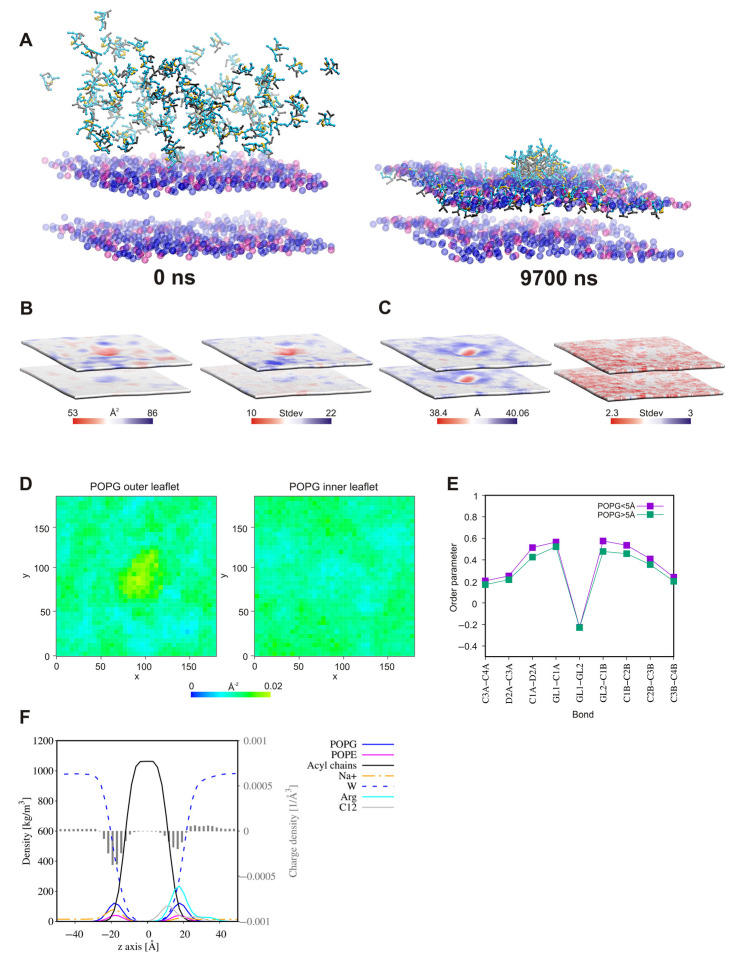
(**A**) Initial and final snapshots from the 3:1 POPG:POPE binding simulations for **B7**. Fatty acid tails are in gray, while arginines are in cyan. Lysine spacers are in orange. Lipid tails were omitted for clarity, and only the POPG and POPE phosphate beads are shown in blue and pink, respectively. (**B**) Local area per lipid (APL) and standard deviations of the local membrane APL and (**C**) local thickness of the bilayer. (**D**) 2D density map of the POPG lipids in the outer and inner leaflets of the POPG:POPE membrane (a grid spacing was set to 5 Å). APL, membrane thickness and 2D density maps were calculated for phosphate beads of the lipid headgroups. (**E**) Lipid acyl chain order parameters of POPG molecules, separately for the lipids at a distance less than and greater than 5 Å from the lipopeptide molecules. (**F**) Partial density and charge density profiles. All the analyses were performed over the last 100 ns of CG MD simulations.

In all the systems, in the initial steps of coarse-grained molecular dynamic simulations (CG MD), the long-range electrostatics draw the positively charged lipopeptides toward the negatively charged membrane surface. Meanwhile, some lipopeptide molecules spontaneously self-assemble into micelles, which are then attracted to the membrane surface. The lipopeptide monomers associated with the membrane surface rapidly enter into the outer membrane leaflet, whereas the surface-bound micelles need more time to penetrate the membrane. Moreover, some of these remain attached to the membrane surface until the end of simulations (Figure 15, Figure 16 and Figure 17). This phenomenon is compatible with conclusions that the oligomers need to overcome a significant free energy barrier to be transferred into membrane [136]. The surface-bound micelles lead to a distinct enrichment of POPG, as well as a decrease in membrane thickness and the area per lipid (APL) at the binding site (Figure 16 and Figure 17). The surfactants increase also the order parameters of the lipid acyl chains located in their close vicinity (Figure 15E, Figure 16E and Figure 17E), the variations appeared to be more pronounced for the system with lipopeptide **B7** acylated with two branched fatty acid chains. This is tantamount to reduction in the mobility of the lipid chains resulting directly from interactions with lipopeptides. Attraction of the positively charged lipopeptides to the negatively charged membrane surface displaces also sodium ions from the outer lipid–water interface and reduces the negative charge density in the outer head-group layer of the membrane (Figure 15F, Figure 16F and Figure 17F). All this indicates that the lipopeptides induce depolarization and permeabilization of the bacterial membrane.

## 3. Materials and Methods

### 3.1. Lipopeptide Synthesis

Compounds were synthesized manually by the solid-phase Fmoc/tBu method, where polystyrene resin modified by Rink Amide linker was used as a solid support (Orpegen Peptide Chemicals GmbH, Heidelberg, Germany). Amino acids used in the study were Fmoc-L-Arg(Pbf)-OH, Fmoc-L-Cys(Trt)-OH, Fmoc-L-Phe-OH (Orpegen Peptide Chemicals GmbH), Fmoc-L-Lys(Fmoc)-OH (Iris Biotech GmbH, Marktredwitz, Germany), and Fmoc-L-Nle-OH (Merck, Darmstadt, Germany). Fatty acids were purchased from Merck (Darmstadt, Germany): 2-butyloctanoic acid [C8(4)], octanoic acid (C8), decanoic acid (C10), dodecanoic acid (C12), and tetradecanoic acid (C14). Deprotection of the Fmoc group was accomplished with a 20% piperidine (Iris Biotech GmbH, Marktredwitz, Germany) solution in *N,N*-dimethylformamide (DMF; POCH, Avantor, Gliwice, Poland) for 15 min. Acylation was performed with a equimolar mixture of N,N′-diisopropylcarbodiimide (DIC), OxymaPure, and Fmoc-AA-OH (or fatty acid) dissolved in DCM:DMF (1:1, *v*/*v*) in fourfold excess based on the resin for 1.5 h (DIC and OxymaPure; Iris Biotech GmbH, Marktredwitz, Germany; DCM; Chempur, Piekary Slaskie, Poland). After deprotection and coupling reactions, the resin was rinsed with DMF and dichloromethane (DCM), and subsequently, the chloranil test was carried out. To obtain gemini surfactants with the l-lysine amide spacer (series **B**), Fmoc-L-Lys(Fmoc)-OH was coupled as a first amino acid to TentaGel^®^ S RAM Resin (loading ca. 0.23 mmol/g; Rapp Polymere GmbH, Tübingen, Germany). Owing to the reduced loading rate of the resin, it was possible to obtain the designed dimers. The remaining compounds (series **A** and **C**) were synthesized using polystyrene resin modified by a Rink Amide linker as a solid support (loading ca. 1.0 mmol/g; Orpegen Peptide Chemicals GmbH, Heidelberg, Germany). Compounds were cleaved from the resin using one of the mixtures (A) trifluoroacetic acid (TFA) (Apollo Scientific, Denton, UK), ) triisopropylsilane TIS (Iris Biotech GmbH, Marktredwitz, Germany), and deionized water (95:2.5:2.5, *v*/*v*/*v*) or (B TFA, TIS, 1,2-ethaneditiol (EDT) (Merck, Darmstadt, Germany), and deionized water (92.5:2.5:2.5:2.5, *v*/*v*/*v*/*v*) for 1.5 h with agitation. Mixture (B) was used with lipopeptides containing cysteine residue and mixture (A) with the remaining surfactants. Then, the compounds were precipitated with cooled diethyl ether (POCH, Avantor, Gliwice, Poland) and lyophilized. The crude lipopeptide with cysteine was dissolved in 20% (*v*/*v*) acetic acid (Chempur, Piekary Slaskie, Poland) solution (0.5 g/L) and oxidized with iodine to obtain a gemini surfactants with intermolecular disulfide bridge (series **C**; l-cystine diamide spacer). The compounds were purified by RP-HPLC. All purifications were carried out on a Waters X-Bridge BEH C18 OBD Prep column (19 × 100 mm, 5 μm particle size, 130 Å pore size). UV detection at 214 nm was used. Compounds were eluted with a linear 20–75% acetonitrile (ACN for HPLC-gradient grade; POCH, Avantor, Gliwice, Poland) gradient in deionized water over 60 min. The mobile phase flow rate was 10.0 mL/min. Both eluents contained 0.1% (*v*/*v*) of TFA. Pure fractions (>95%, HPLC) were collected and lyophilized. The identity of all compounds was confirmed by mass spectrometry (ESI-MS; Waters Alliance e2695 system with Acquity QDa detector; Waters, Milford, MA, USA).

### 3.2. Determination of Lipopeptide Hydrophobicity with RP-HPLC

Lipopeptide hydrophobicity was determined as described previously [120]. To do this, a Waters Alliance e2695 system with a Waters 2998 PDA Detector (software-Empower 3, Waters, Milford, MA, USA) was used. All analyses were carried out on a Waters X-Bridge Shield RP-18 column (3.0 × 100 mm, 3.5 μm particle size, 130 Å pore size). The Shield Technology column with embedded polar groups was used to minimize interactions of unreacted silanol groups with basic lipopeptides [137]. The peptides were dissolved in water (0.1% TFA, *v*/*v*) up to a concentration of 1 mg/mL. UV detection at 214 nm was used, and samples (10 μL) were eluted with a linear 20–65% acetonitrile gradient in deionized water over 30 min at 25.0 ± 0.1 °C. The mobile phase flow rate was 0.5 mL/min. Both eluents contained 0.1% (*v*/*v*) of TFA. Each peptide sample was analyzed in triplicate. The maximum standard deviation and coefficient of variation were 0.044 and 0.85%, respectively.

### 3.3. Critical Aggregation Concentration Measurements

The critical aggregation concentration (CAC) values were determined fully automatically using a K100 tensiometer (KRUSS, Hamburg, Germany) equipped with two micro dosimeters by measuring the surface tension vs. lipopeptide concentration. The measurements were performed in unbuffered aqueous solution at 298 K using the Du Noüy ring method. The accuracy of the surface tension measurements was 0.1 mN/m. The critical aggregation concentration (CAC) was defined as the intersection of two lines fitted to the experimental data collected before and after the inflection point.

### 3.4. Antimicrobial Activity

#### 3.4.1. Cultivation of Microorganisms

The *Acinetobacter baumannii* ATCC BAA-1605, *Enterococcus faecium* ATCC 700221, *Escherichia coli* ML-35 ATCC 43827, *Klebsiella aerogenes* ATCC 13048, *Klebsiella pneumoniae* ATCC 700603, *Pseudomonas aeruginosa* ATCC 9027, *Staphylococcus aureus* ATCC 25923, *Staphylococcus aureus* ATCC 33591, *Candida albicans* ATCC 10231, *Candida glabrata* ATCC 15126, strains were acquired from the American Type Culture Collection (ATCC). All the strains were stored at −80 °C in Roti-Store cryo vials and before the tests were transferred into fresh Mueller–Hinton broth (MHB, Biocorp, Warsaw, Poland) for bacteria or RPMI-1640 (Sigma-Aldrich, Steinheim, Germany) for fungi and incubated for 24 h at 37 °C. Then, the cultures were seeded on the Mueller–Hinton agar (BioMaxima, Lublin, Poland) or Sabouraud dextrose agar (SDA, BioMaxima, Lublin, Poland) plates, respectively, and incubated as just mentioned. These agar cultures were used for further microbiological assays. Cell densities for all assays were adjusted spectrophotometrically (Multiskan GO Microplate Spectrophotometer, Thermo Fisher Scientific, Vantaa, Finland) at 600 and 530 nm for bacteria and fungi, respectively.

#### 3.4.2. Activity against Planktonic Cultures

The MICs were determined by the broth microdilution method according to the Clinical and Laboratory Standard Institute guidelines [138,139]. For this purpose, initial inoculums of bacteria (5 × 10^5^ CFU/mL) in MHB and yeasts (2 × 10^3^ CFU/mL) in RPMI-1640 with 2% D-glucose were exposed to the ranging concentration of lipopeptides (0.5–256 μg/mL) and incubated at 37 °C for 18 h and 24 h, respectively. The experiments were conducted on 96-well microtiter polystyrene plates (Kartell, Noviglio, Italy). The growth was assessed visually after incubation, and the MIC was assumed as the lowest peptide concentration at which a noticeable growth of microorganisms was inhibited. All experiments were conducted in triplicate.

#### 3.4.3. Activity against Biofilm

The MBECs were determined as described previously using 96-well polystyrene flat-bottom plates [15,120]. For this purpose, 24 h cultures of microorganisms were diluted to obtain a final density of 5.0 × 10^5^ CFU/mL and 2.0 × 10^5^ CFU/mL of bacteria and fungi, respectively. Microorganisms were diluted in MHB or RPMI-1640 with 2% D-glucose (bacteria and fungi, respectively). Briefly, 100 µL of cell suspension was added into the test plates. After 24 h of incubation at 37 °C, the wells were rinsed three times with a PBS (pH 7.4) to remove non-adherent cells. Subsequently, 100 μL of the test compounds in a concentration range (1–512 μg/mL) were added to each well. After 24 h of incubation at 37 °C, 20 μL of a cell viability reagent was added (resazurin, 4 g/L; Sigma Aldrich, St. Louis, MO, USA). The MBEC was read after 1 h. The determined values were recorded as the lowest concentration at which the reduction of resazurin (from blue to pink) was lower or equal to 10 ± 0.5% as compared to the positive (100%) and negative (0%) controls. The reduction was monitored by measuring absorbance at 570 nm (reduced) and 600 nm (oxidized) using a microplate spectrophotometer (Multiskan GO Microplate Spectrophotometer, Thermo Fisher Scientific, Vantaa, Finland). All experiments were conducted in triplicate.

#### 3.4.4. Antimicrobial Activity in the Presence of Serum

The MICs were determined in the presence of normal human serum (NHS; Sigma-Aldrich, Human Serum, Normal) in concentrations of 1 and 10% (*v*/*v*). NHS was stored frozen at −20 °C and thawed just before use. Microorganisms were cultivated as described in 3.4.1. Serial dilutions of compounds, inoculum density, medium, and incubation conditions were prepared as described in Section 3.4.2. Stock solutions of test compounds were prepared in appropriate culture medium.

### 3.5. Hemolysis Assay

The hemolysis assay was performed by using the method reported previously [15,140]. Fresh human red blood cells (hRBCs) with anticoagulant (EDTA) were rinsed three times with a PBS by centrifugation at 800× *g* for 10 min and diluted with PBS. The compounds were serially diluted on a 96-well microtiter polystyrene plate, and hRBCs were added up to a final volume of 100 μL. The compound concentration ranged between 0.5 and 256 μg/mL and the final hRBCs concentration was 4% (*v*/*v*). Controls for zero (blank) and 100% hemolysis consisted of hRBCs suspended in PBS and 1% of Triton-X 100 (Carl Roth GmbH, Karlsruhe, Germany), respectively. The plate was incubated for 1 h at 37 °C and then centrifuged (800× *g*, 10 min, 4 °C). Subsequently, the supernatant was resuspended to new microtiter plates, and the absorbance at 540 nm was measured (Multiskan GO Microplate Spectrophotometer, Thermo Fisher Scientific, Vantaa, Finland). All experiments were conducted in triplicate. HC_50_ was calculated using an ic50.tk tool. The protocol of the study was approved by the local Bioethics Committee at the Medical University of Gdańsk (NKBBN/262/2019, approval date: 10 June 2019).

### 3.6. MTS Assay

The human keratinocytes HaCaT cell line (Elabscience^®^, Houston, TX, USA) was cultured in DMEM-high glucose (Dulbecco’s Modified Eagle Medium) supplemented with fetal bovine serum (10%), penicillin–streptomycin (1%) (Sigma Aldrich/Merck), and incubated at 37 °C under a humidified atmosphere of 5% CO_2_. Cells were trypsinized, and the medium was renewed 2–3 times per week. The cytotoxicity of compounds was assessed by the determination of cell metabolic activity by colorimetric CellTilter 96^®^ Aqueous One Solution Cell Proliferation Assay (Promega, Madison, WI, USA), according to the manufacturer’s instructions. In brief, 7 × 10^3^ of HaCaT cells per well were plated on 96-well plates and incubated per night to adhere to the cultured dishes. After that time, different concentrations of the tested compounds ranging from 500 to 1.7 μg/mL were applied to the wells. Every compound was medium-soluble in 37 °C; thus, the control cells were cultured with the medium. Serial dilutions of the tested compounds were always prepared prior to use. Cells were incubated with drugs for 24 h, and then, 20 μL of the CellTiter 96^®^AqueousOne Solution Reagent was added to each well containing cells maintained in 100 μL of culture medium. After 4 h of incubation at 37 °C, the absorbance at 490 nm was recorded using a microplate reader (Epoch, BioTek Instruments, Winooski, VT, USA). At least three independent experiments with two replicates were conducted for each concentration of compound, and the IC_50_ values were calculated using GraphPad Prism 8 software.

### 3.7. Bacterial Membrane Permeabilization Assays

Inner and outer membranes permeabilization kinetics were evaluated using *Escherichia coli* ML-35 (ATCC 43827) and chromogenic substrates—CENTA (BioVision, Milpitas, CA, USA) and o-nitrophenyl-β-d-galactopyranoside (ONPG) (Sigma-Aldrich, Steinheim, Germany), as described previously [120]. To examine OM permeabilization, CENTA was used as a β-lactamase substrate. In effect of OM permeabilization, the enzyme can hydrolyze CENTA’s β-lactam ring. The resulting color change (2-nitro-5-thiobenzoate anion, TNB^-^) could be measured spectrophotometrically at 405 nm [141]. The IM permeabilization was monitored with ONPG, which is a chromogenic β-galactosidase substrate. The product of this reaction (4-nitrophenol) was measured spectrophotometrically at 405 nm (Multiskan GO Microplate Spectrophotometer, Thermo Fisher Scientific, Vantaa, Finland). Compounds concentrations in OM and IM permeabilization assays were MIC (32 μg/mL of **A4**, **B2**, **C2**, **B7** and 64 μg/mL of **C7**). Bacteria were incubated in lysogeny broth medium (LB; Biocorp, Warszawa, Poland) for 24 h at 37 °C. Subsequently, the cells were diluted in a fresh LB medium and incubated at 37 °C up to a mid-log phase (ca. 3 h). The culture was centrifuged (3 min, 1100× *g*) and rinsed twice with a PBS. The bacteria were resuspended in PBS up to a concentration of 5 × 10^6^ CFU/mL. The final concentration of ONPG and CENTA was 1.5 mM and 0.15 mM, respectively. The ONPG/CENTA in PBS (Sigma-Aldrich, Steinheim, Germany) was used as a negative control. Polymyxin B sulfate (Sigma-Aldrich, Steinheim, Germany) in PBS at MIC (2 µg/mL) was used as a positive control. Readings were taken every 15 min for 3 h at 37 °C [125,142,143,144]. Experiments were performed in triplicate on 96-well flat bottom microtiter polystyrene plates.

### 3.8. Serum Stability

#### 3.8.1. Incubation in Serum

Serum stability of peptides **B7** and **C7** was tested in normal human serum (Sigma-Aldrich, Steinheim, Germany) according to the modified Jenssen and Aspmo protocol [145]. Briefly, compounds were incubated in 25% (*v*/*v*) human serum solution in RPMI-1640 (Sigma-Aldrich, Steinheim, Germany) at 37 ± 0.1 °C. The final compound concentration was 50 µg/mL. Time intervals were 0, 1, 2, 3, 4, and 5 h. To precipitate serum proteins, 100 µL of solution was added to 200 µL of 96% ethanol (Pure P.A., POCH, Avantor Performance Materials S.A, Gliwice, Poland) and incubated at 4 °C before centrifugation (4 °C, 18,000× *g*, 2 min; Sorvall ST 16R Centrifuge, Thermo Scientific, Osterode am Harz, Germany). Subsequently, 200 µL of supernatant was transferred to glass vials (1.5 mL, Anchem, Poland), and the liquid was evaporated under nitrogen at 37 °C (approximately 30 min). The vials were lyophilized for 24 h to remove traces of solvents.

#### 3.8.2. Calibration Curve

The influence of serum matrix on calibration curves was eliminated by the preparation of a series of vials with residual substances from serum. Briefly, ethanol was added to serum solution to precipitate proteins. Subsequently, the precipitate was centrifuged, and the supernatant was collected and evaporated to dryness as described above. Standards of the analyzed compounds were prepared in demineralized water in concentration ranging from 1 to 15 ppm. Next, 333 µL of solutions were moved to vials containing residual matrix substances (blank, without surfactant). Similarly, samples obtained after serum stability experiments were dissolved in 333 µL of demineralized water and analyzed. Analyses were performed using UPLC-HRMS. The mobile phase contained deionized water (solvent A) and acetonitrile (solvent B; ACN for HPLC-gradient grade; POCH, Avantor, Gliwice, Poland) both supplemented with a 0.1% (*v*/*v*) formic acid (Sigma-Aldrich, Steinheim, Germany).

#### 3.8.3. UPLC-HRMS Analytical Parameters

The quantification of lipopeptides was performed using an Agilent 1290 Infinity chromatograph and Agilent 6550 Quadrupole Time-of-flight (Q-TOF) high-resolution mass spectrometer (HRMS). Gradient LC analyses were conducted using a ZORBAX Eclipse Plus C18 column (Rapid Resolution HD, 2.1 × 50 mm, 1.8 µm) and mobile phases: A—water containing 0.3% of acetonitrile and B—acetonitrile, both with 0.1% (*v*/*v*) formic acid. Separation was achieved in a linear gradient program 20–98% of B over 5 min with 0.4 mL/min flow at 24.0 ± 0.1 °C. The mass detector equipped with an ESI source in HRMS mode was set in scanning mode 100–1700 m/z range in positive ionization. Chromatograms for a desired m/z value were extracted, and calibration curves were prepared using Mass Hunter software. All standards and serum samples were subjected to LC-MS analysis, and all analyses were performed in triplicate. Calibration curves were prepared for chromatograms extracted for m/z values corresponding to mono-, di-, tri- and tetra-charged ions. For analysis of peptide **C7**, a pseudomolecular ion [M+H]^+^ (*m*/*z* 1227.8017) was selected, while for the other peptide **B7**, triple charged ion [M+3H]^3+^ (*m*/*z* 378.9606).

### 3.9. Molecular Dynamics Simulations

All-atom molecular dynamic simulations were used to study the self-assembly tendency of the lipopeptides. The simulations were performed using Graphics Processing Unit/Compute Unified Device Architecture (GPU/CUDA)-accelerated of Particle Mesh Ewald Molecular Dynamics (PMEMD) in Assisted Model Building with Energy Refinement (AMBER) 16 package [146]. Non-standard residues were modeled with a LEaP module of AMBER 16. The point charges were optimized by fitting them to the ab initio molecular electrostatic potential (6-31G* basis set, GAMESS 2013—ab initio molecular electronic structure program) [147] for two different conformations of each non-standard residue. The final charges were averaged over both conformations, as recommended by the Restrained electrostatic potential (RESP) protocol [148]. The tools borrowed from the GROMACS 2019 package (gmx insert-molecules and solvate) [149] were used to build initial configurations of randomly placed lipopeptides in a water box (130 Å × 130 Å × 130 Å). Chloride ions were added to neutralize the lipopeptide molecules with the LEaP module of the AMBER 16. The systems were minimized for 40,000 steps (steepest descent method) with weak positional harmonic constraints (10 kcal mol^−1^ Å^−1^) on the peptide molecules for the first 10,000 steps. Subsequently, the system was equilibrated using a three-step protocol: (1) 10 ns simulations with isotropic pressure coupling and 0.5 fs time step, (2) 10 ns simulation with isotropic pressure coupling and 1 fs time step, and (3) 500 ns simulations with anisotropic pressure coupling and 2 fs time step. The long-range electrostatic interactions were evaluated by the particle Mesh Ewald (PME) summation. A cut-off of 10 Å was used for van der Waals interactions. The SHAKE algorithm was used to constrain bonds involving hydrogen. The temperature was maintained using the Langevin coupling scheme, whereas a Berendsen barostat maintained a reference pressure set to 1.0 bar.

The MD simulations of surfactant–membrane interactions were carried out in the MARTINI coarse-grained force field [150,151] implemented in the GROMACS 2019 package [149]. The CHARMM-GUI web-based graphical interface [152,153,154,155] was used to build a bilayer model of Gram-positive bacteria composed of 740 POPG and 246 POPE equally distributed between two membrane leaflets. The membrane with sodium ions neutralizing POPG lipids but without a water box was used to construct the systems with the lipopeptides. Surfactant molecules were randomly placed close to the outer leaflet of the membrane. The systems were solvated, and the lipopeptides were neutralized by chloride ions using the standard GROMACS tools. The salt concentration for bulk solution was kept at a 100 mM NaCl. Each system was energy-minimized and equilibrated with the stepwise lowered force constant of the harmonic restraints (from 200 to 10 kJ mol^−1^ nm^−2^) to fix the position of the headgroups of the lipids during simulations. After equilibration, the systems were subjected to isothermal–isobaric molecular dynamics (NTP, Normal Temperature and Pressure) with a 10 fs time step [156]. The pressure was treated isotropically at 1 bar using the Parinello–Rahman barostat with a coupling constant τp = 12.0 ps. The temperature was held at 310 K using the Nose–Hoover temperature coupling. The relative dielectric constant for explicit screening was 15. Coulomb interactions were treated using a reaction-field and a cutoff of 11 Å. Due to a high concentration of lipopeptides near the membrane surface, some of the lipopeptides were free to move away and bound to the inner membrane leaflet as a consequence of periodic boundary conditions. These surfactant molecules along with close counterions were removed from the systems to maintain the natural state where the lipopeptides were only able to interact with the outer leaflet of the membrane in the initial steps of interactions. After this correction, the CG MD simulations were continued for at least 7 µs.

The area per lipid (APL) and membrane thickness were calculated using the GROMACS compatible analysis tool g_lomepro [157]. The order parameters for Martini lipids were calculated with a do-ordered-gmx5.py script available from the Martini website (www.cgmartini.nl, accessed on 11 May 2020). All visualizations were created with VMD [158].

## 4. Conclusions

This article has been focused on the biological and physico-chemical characteristics of arginine-rich gemini surfactants and their monomeric parent molecules (series **A**). Geminis with two different spacers have been studied, namely with l-lysine amide (series **B**) and l-cystine diamide (series **C**). Moreover, N-terminal hydrophobic amino acids (norleucine and phenylalanine), straight-chain as well as branched fatty acids were used. The results provide evidence that gemini compounds exhibit distinctly higher antimicrobial activities than the corresponding monomers against both planktonic and biofilm cultures. It is noteworthy that these compounds show an elevated selectivity between pathogens and normal human cells as compared to those of monomers and are thus promising candidates for further in vivo studies. Interestingly, surfactants with branched fatty acids were found to be alternatives to straight-chain ones owing to their excellent biological characteristics. Their antimicrobial activity was the least affected by human serum. Interestingly, the gemini compound with the l-cystine diamide spacer exhibited comparable serum stability to that of the analogous surfactant with the l-lysine amide spacer. In some cases, dimers with norleucine or phenylalanine residues were more efficient antimicrobials with a slightly higher selectivity to planktonic cells of *P. aeruginosa* and *K. pneumoniae* over human cells than geminis with straight-chain fatty acids of comparable hydrophobicity. Molecular dynamics and membrane permeabilization kinetics studies supported the hypothesis that geminis effectively interact with bacterial membranes. In general, the LogCAC of surfactants was linearly correlated with hydrophobicity. However, the branched-chain fatty acid seems to stimulate a reduced tendency to self-aggregation seemingly due to the relatively high CAC and the results of MD analysis. Some of the compounds should be evaluated in further in vivo studies owing to their high antimicrobial activity and low-to-moderate cytotoxicity, e.g., **B2** and **B7** in a model of *S. aureus* skin infection or **B6** and **C7** in a model of *P. aeruginosa* infection. Moreover, various branched-chain fatty acids should be involved in further systematic studies on gemini surfactants to learn how different branches and chain lengths can affect the biological and physico-chemical characteristics of surfactants.

## Figures and Tables

**Table 1:** Physico-chemical properties of lipopeptides.

Peptide	Lipopeptide Fragment	Spacer	Adjusted Retention Time (min)	CAC mM (μg/mL)	Surface Tension at CAC (mN/m)
**A1** ^a^	C8-RR-NH_2_	-	2.93	nd	nd
**A2** ^a^	C10-RR-NH_2_	-	7.74	nd	nd
**A3**	C12-RR-NH_2_	-	12.15	7.48 (3827.3)	38
**A4**	C14-RR-NH_2_	-	16.31	1.67 (899.7)	35
**B1** ^a^	C8-RR-	l-Lysine amide	8.11	nd	nd
**B2**	C10-RR-	l-Lysine amide	13.73	3.69 (3980.0)	36
**B3**	C12-RR-	l-Lysine amide	19.09	0.87 (987.3)	41
**B4**	C14-RR-	l-Lysine amide	25.33	0.31 (365.7)	40
**B5**	C8-FRR-	l-Lysine amide	15.19	2.47 (3247.9)	39
**B6**	C8-NleRR-	l-Lysine amide	13.96	3.59 (4482.4)	37
**B7** ^b^	C8(4)-RR-	l-Lysine amide	14.68	nd	nd
**C1** ^a^	C8-RR-	l-Cystine diamide	8.26 (0.15) ^c^	nd	nd
**C2**	C10-RR-	l-Cystine diamide	13.94 (0.21) ^c^	3.30 (3832.7)	43
**C3**	C12-RR-	l-Cystine diamide	19.24 (0.15) ^c^	0.25 (305.4)	43
**C4**	C14-RR-	l-Cystine diamide	25.30 (−0.03) ^c^	0.06 (71.1)	47
**C5**	C8-FRR-	l-Cystine diamide	15.42 (0.23) ^c^	2.58 (3632.7) ^d^	37
**C6**	C8-NleRR-	l-Cystine diamide	14.29 (0.33) ^c^	4.28 (5737.7)	35
**C7**	C8(4)-RR-	l-Cystine diamide	14.83 (0.15) ^c^	6.21 (7628.1)	35

^a^ Critical aggregation concentrations (CAC) value was not determined due to the high CAC value and consequently large amount of the peptide required to prepare initial solution; ^b^ The CAC value was not determined due to a poor solubility of the peptide at a higher concentration. The 9.76 mM solution reduced the surface tension to 34 mM/m, but the CAC was not reached; ^c^ Difference in retention time calculated as tR’C—tR’B (between corresponding surfactants e.g., **C1** and **B1**); ^d^ The initial solutions with concentrations above CAC were cloudy.

**Table 2:** Hemolysis (HC_50_), cytotoxicity (IC_50_), and antimicrobial activity against planktonic cultures. The selectivity indexes (SI) human red blood cells (hRBCs) and immortalized human keratinocytes cell line SI HaCaT are in brackets on the left and right, respectively.

Compound	*HC_50_*	*IC_50_*	*Enterococcus faecium* ATCC 700221	*Staphylococcus aureus* ATCC 33591	*Staphylococcus aureus* ATCC 25923	*Klebsiella pneumoniae* ATCC 700603	*Acinetobacter baumannii* ATCC BAA 1605	*Pseudomonas aeruginosa* ATCC 9027	*Klebsiella aerogenes* ATCC 13048	*C. albicans* ATCC 10231	*C. glabrata* ATCC 15126
**A1**	>256 *	>500	>256 (−/−)	>256 (−/−)	>256 (−/−)	>256 (−/−)	>256 (−/−)	>256 (−/−)	>256 (−/−)	>256 (−/−)	>256 (−/−)
**A2**	>256 *	>500	256 (−/−)	256 (−/−)	256 (−/−)	>256 (−/−)	>256 (−/−)	>256 (−/−)	>256 (−/−)	>256 (−/−)	>256 (−/−)
**A3**	>256 *	153.50 ± 8.13	32 (>8/4.80)	32 (>8/4.80)	32 (>8/4.80)	>256 (−/−)	256 (−/−)	128 (>2/1.20)	>256 (−/−)	64 (>4/2.40)	128 (>2/1.20)
**A4**	68.43 ± 1.41 *	46.48 ± 3.65	8 (6.02/5.81)	8 (6.02/5.81)	8 (6.02/5.81)	256 (−/−)	64 (0.75/0.73)	32 (1.50/1.45)	256 (−/−)	8 (6.02/5.81)	16 (3.01/2.91)
**B1**	>256	378.40 ± 93.71	64 (>4/5.91)	8 (>32/47.30)	4 (>64/94.60)	>256 (−/−)	256 (−/−)	32 (>8/11.83)	>256 (−/−)	64 (>4/5.91)	64 (>4/5.91)
**B2**	>256	150.03 ± 3.15	2 (>128/75.02)	1 (>256/150.03)	1 (>256/150.03)	128 (>2/1.17)	8 (>32/18.75)	8 (>32/18.75)	128 (>2/1.17)	8 (>32/18.75)	4 (>64/37.51)
**B3**	56.29 ± 6.50	26.42 ± 4.24	2 (28.15/13.21)	2 (28.15/13.21)	4 (14.07/6.61)	64 (0.88/0.41)	8 (7.04/3.30)	4 (14.07/6.61)	32 (1.76/0.83)	2 (28.15/13.21)	2 (28.15/13.21)
**B4**	44.12 ± 4.70	32.39 ± 4.49	8 (5.52/4.05)	64 (0.69/0.51)	64 (0.69/0.51)	32 (1.38/1.01)	64 (0.69/0.51)	32 (1.38/1.01)	32 (1.38/1.01)	4 (11.03/8.10)	2 (22.06/8.10)
**B5**	181.38 ± 8.99	169.13 ± 32.80	4 (45.35/42.28)	4 (45.35/42.28)	2 (90.69/84.57)	128 (1.42/1.32)	32 (5.69/5.29)	8 (22.67/21.14)	256 (−/−)	16 (11.34/10.57)	16 (11.34/10.57)
**B6**	>256	164.17 ± 14.65	2 (>128/82.09)	4 (>64/41.04)	2 (>128/82.09)	64 (>4/2.57)	32 (>8/5.13)	4 (>64/41.04)	256 (−/−)	16 (>16/10.26)	16 (>16/10.26)
**B7**	>256	78.78 ± 6.93	1 (>256/78.78)	0.5 (>512/157.56)	1 (>256/78.78)	32 (>8/2.46)	32 (>8/2.46)	4 (>64/19.70)	16 (>16/4.92)	8 (>32/9.85)	16 (>16/4.92)
**C1**	>256	>500	32 (>8/>15.63)	16 (>16/>31.25)	8 (>32/>62.50)	>256 (−/−)	256 (−/−)	64 (>4/>7.81)	>256 (−/−)	128 (>2/>3.91)	64 (>4/>7.81)
**C2**	>256	231.17 ± 24.37	2 (>128/115.59)	1 (>256/231.17)	2 (>128/115.59)	>256 (−/−)	16 (>16/14.45)	8 (>32/28.90)	128 (>2/1.81)	4 (>64/57.79)	8 (>32/28.90)
**C3**	36.24 ± 2.35	119.47 ± 6.03	2 (18.12/59.74)	4 (9.06/29.87)	4 (9.06/29.87)	>256 (−/−)	64 (0.57/1.87)	8 (4.53/14.93)	32 (1.13/3.73)	1 (36.24/119.47)	2 (18.12/59.74)
**C4**	20.89 ± 1.06	54.52 ± 15.40	8 (2.61/6.82)	32 (0.65/1.70)	64 (0.33/0.85)	>256 (−/−)	32 (0.65/1.70)	64 (0.33/0.85)	128 (0.16/0.43)	2 (10.45/27.26)	1 (20.89/54.52)
**C5**	117.06 ± 4.60	146.63 ± 14.95	2 (58.53/73.32)	2 (58.53/73.32)	2 (58.53/73.32)	32 (3.66/4.58)	32 (3.66/4.58)	8 (14.63/18.33)	128 (0.91/1.15)	8 (14.63/18.33)	8 (14.63/18.33)
**C6**	>256	248.83 ± 18.81	2 (>128/124.42)	2 (>128/124.42)	2 (>128/124.42)	64 (>4/3.89)	32 (>8/7.78)	8 (>32/31.10)	256 (−/−)	16 (>16/15.55)	16 (>16/15.55)
**C7**	>256	163.98 ± 13.66	2 (>128/81.99)	2 (>128/81.99)	2 (>128/81.99)	32 (>8/5.12)	32 (>8/5.12)	4 (>64/41.00)	16 (>16/10.25)	16 (>16/10.25)	16 (>16/10.25)

* These analyses were performed by our group in the previous study [15].

**Table 3:** Calculated LogP values for series **C** and the corresponding monomers. cLogPs were calculated with ACD/ChemSketch 2020.1.2.

Code	cLogP (Dimer)	cLogP (Corresponding Monomer)
**C1**	−0.05 ± 0.92	−0.43 ± 0.75
**C2**	2.07 ± 0.92	0.64 ± 0.75
**C3**	4.20 ± 0.92	1.70 ± 0.75
**C4**	6.32 ± 0.92	2.76 ± 0.75
**C5**	3.09 ± 0.97	1.15 ± 0.85
**C6**	2.60 ± 0.96	0.90 ± 0.82
**C7**	3.83 ± 0.93	1.52 ± 0.75

**Table 4:** Antimicrobial activity of lipopeptides in the presence of human serum.

MIC (µg/mL)									
Strain	*S. aureus* ATCC 33591			*P. aeruginosa* ATCC 9027			*C. glabrata* ATCC 15126		
Serum Concentration % (*v*/*v*)	0%	1%	10%	0%	1%	10%	0%	1%	10%
**A1**	>256	>256	>256	>256	>256	>256	>256	>256	>256
**A2**	256	>256	>256	>256	>256	>256	>256	>256	>256
**A3**	32	64	128	128	256	>256	128	256	>256
**A4**	8	16	64	32	128	256	16	64	256
**B1**	8	32	>256	32	>256	>256	64	>256	>256
**B2**	1	32	64	8	64	256	4	64	>256
**B3**	2	8	16	4	32	64	2	16	>256
**B4**	64	16	64	32	128	>256	2	32	>256
**B5**	4	64	>256	8	128	>256	16	64	256
**B6**	4	128	>256	4	256	>256	16	128	>256
**B7**	0.5	4	8	4	64	128	16	32	64
**C1**	16	>256	>256	64	>256	>256	64	>256	>256
**C2**	1	64	>256	8	128	>256	8	128	>256
**C3**	4	32	32	8	64	256	2	32	>256
**C4**	32	16	64	64	128	256	1	32	>256
**C5**	2	64	256	8	128	>256	8	128	>256
**C6**	2	128	>256	8	256	>256	16	128	>256
**C7**	2	32	64	4	128	256	16	64	256

**Table 5:** Antimicrobial activity against biofilm.

Compound	*Enterococcus faecium* ATCC 700221	*Staphylococcus aureus* ATCC 33591	*Staphylococcus aureus* ATCC 25923	*Klebsiella pneumoniae* ATCC 700603	*Acinetobacter baumannii* ATCC BAA 1605	*Pseudomonas aeruginosa* ATCC 9027	*Klebsiella aerogenes* ATCC 13048	*Candida albicans* ATCC 10231	*Candida glabrata* ATCC 15126
**A1**	>512	>512	>512	>512	>512	>512	>512	>512	>512
**A2**	512	512	512	>512	>512	>512	>512	512	>512
**A3**	32	64	64	512	512	512	512	128	512
**A4**	8	8	16	256	256	512	256	64	256
**B1**	128	32	32	>512	512	512	>512	256	>512
**B2**	4	4	8	256	128	512	256	32	512
**B3**	16	16	64	128	256	512	128	32	64
**B4**	64	128	256	512	512	>512	256	64	64
**B5**	8	4	16	256	256	512	256	32	256
**B6**	8	4	16	256	256	256	256	256	512
**B7**	8	4	8	128	64	256	64	64	256
**C1**	64	32	64	>512	512	512	>512	256	>512
**C2**	4	8	32	512	256	512	256	32	512
**C3**	16	32	128	256	512	512	256	16	128
**C4**	64	128	512	512	512	>512	512	16	64
**C5**	8	8	64	256	512	512	256	128	256
**C6**	8	8	32	256	512	512	256	256	512
**C7**	4	8	32	256	256	512	128	64	128

## Data Availability

Data are reported in the text and in supplementary files.

## Supplementary Materials

The following are available online at https://www.mdpi.com/1422-0067/22/7/3299/s1, Figure S1: Chemical structure of B5, Figure S2: Chemical structure of B6, Figure S3: Chemical structure of B7, Figure S4: Chemical structure of C5, Figure S5: Chemical structure of C6, Figure S6: Chemical structure of C7, Figure S7: IM permeabilization kinetics of C2, Figure S8: Log_2_(C2 concentration) vs. absorbance at 405 nm at 240 min, Figure S9: Exemplary results of membrane kinetics studies, Figure S10: Log_2_(C2 concentration) vs. t_i_ at different concentrations of C2, Table S1: MS analyses and net charge, Table S2: Percentage of B7 and C7 remaining upon incubation with normal human serum.

## Abbreviations

ACN	acetonitrile
AMPs	antimicrobial peptides
APL	area per lipid
ATCC	American Type Culture Collection
AU	absorbance unit
BSA	bovine serum albumin
CAC	critical aggregation concentration
CENTA	(6R,7R)-3-{[(3-Carboxy-4-nitrophenyl)sulfanyl]methyl}-8-oxo-7-[(2-thienylacetyl)amino]-5-thia-1-azabicyclo [4.2.0]oct-2-ene-2-carboxylic acid
CFU	colony forming units
CLSI	Clinical and Laboratory Standards Institute
CMC	critical micelle concentration
DCM	dichloromethane
DIC	*N,N*′-diisopropylcarbodiimide
DMF	*N,N*-dimethylformamide
EDT	1,2-ethaneditiol
EDTA	ethylenediaminetetraacetic acid
ESI	electrospray-ionization
Fmoc	9-fluorenylmethoxycarbonyl
GSH	reduced glutathione
HaCaT	immortalized human keratinocytes cell line
HC_50_	surfactant concentration causing 50% hemolysis
hRBCs	human red blood cells
HSA	human serum albumin
IC_50_	surfactant concentration causing 50% inhibition of the growth
IM	inner membrane
LC-MS	liquid chromatography-mass spectrometry
MBEC	minimum biofilm eradication concentration
MD	molecular dynamics
MHB	Mueller–Hinton broth
MIC	minimum inhibitory concentration
MRSA	methicillin-resistant *Staphylococcus aureus*
MTS	4-{5-[3-(Carboxymethoxy)phenyl]-3-(4,5-dimethyl-1,3-thiazol-2-yl)-2H-tetrazol-3-ium-2-yl}benzenesulfonate; tetrazolium inner salt
nd	not determined
NHS	normal human serum
OM	outer membrane
ONP	ortho-nitrophenol
ONPG	o-nitrophenyl-β-d-galactopyranoside
OxymaPure	ethyl (2E)-cyano(hydroxyimino)acetate
PBS	phosphate-buffer saline
POPE	1-palmitoyl-2-oleoyl-sn-glycero-3-phosphoethanolamine
POPG	1-palmitoyl-2-oleoyl-sn-glycero-3-phosphoglycerol
Q-TOF	quadrupole time-of-flight
RP-HPLC	reverse-phase high-performance liquid chromatography
SDA	Sabouraud dextrose agar
SI	selectivity index
tBu	*tert*-butyl
TFA	trifluoroacetic acid
TIS	triisopropylsilane
TNB^-^	2-nitro-5-thiobenzoate anion
UPLC-HRMS	ultra-performance liquid chromatography-high-resolution mass spectrometry
USCLs	ultrashort cationic lipopeptides
VRE	vancomycin-resistant enterococci
WHO	World Health Organization
