# Supplementary material for: Biological and Physico-Chemical Characteristics of Arginine-Rich Peptide Gemini Surfactants with Lysine and Cystine Spacers
Source: Int J Mol Sci. 2021 Mar 24;22(7):3299. doi: 10.3390/ijms22073299 (PMC8036666; doi:10.3390/ijms22073299)
Supplement: Supplementary file 1 [file ijms-22-03299-s001.pdf]

Supplementary Materials

Biological and physico-chemical characteristics of arginine-rich peptide gemini surfactants with lysine and cystine spacers

Table S1. MS analyses and net charge.

**Code**	**Lipopeptide fragment**	**Spacer**	**Av. Mass (Da)**	**Mono-isotopic Mass (Da)**	**MS Analysis**			**Net charge**
					**z**[a]	***m/z* Calc.**	***m/z* Found**	
**A1**	C8-RR-NH2	-	455.60	455.33	1<br>2	456.34<br>228.67	456.41<br>228.78	+2
**A2**	C10-RR-NH2	-	483.65	483.36	1<br>2	484.37<br>242.69	484.48<br>242.78	+2
**A3**	C12-RR-NH2	-	511.70	511.40	1<br>2	512.40<br>256.71	512.47<br>256.85	+2
**A4**	C14-RR-NH2	-	539.76	539.43	1<br>2	540.44<br>270.72	540.52<br>270.87	+2
**B1**	C8-RR-	L-Lysine amide	1022.34	1021.73	1<br>2<br>3<br>4	1022.74<br>511.88<br>341.59<br>256.44	-<br>512.00<br>341.65<br>256.47	+4
**B2**	C10-RR-	L-Lysine amide	1078.44	1077.80	1<br>2<br>3<br>4	1078.81<br>539.91<br>360.27<br>270.46	-<br>540.25<br>360.54<br>270.63	+4
**B3**	C12-RR-	L-Lysine amide	1134.55	1133.86	1<br>2<br>3<br>4	1134.87<br>567.94<br>378.96<br>284.47	1134.94<br>568.23<br>379.26<br>284.64	+4
**B4**	C14-RR-	L-Lysine amide	1190.66	1189.92	1<br>2<br>3<br>4	1190.93<br>595.97<br>397.65<br>298.49	-<br>596.37<br>397.99<br>298.76	+4
**B5**	C8-FRR-	L-Lysine amide	1316.69	1315.87	1<br>2<br>3<br>4	1316.88<br>658.94<br>439.63<br>329.98	-<br>659.37<br>440.12<br>330.58	+4
**B6**	C8-NleRR-	L-Lysine amide	1248.65	1247.90	1<br>2<br>3	1248.91<br>624.96<br>416.98	-<br>625.40<br>417.37	+4

					4	312.98	313.37	
**B7**[b]	C8(4)-RR-	L-Lysine Amide	1134.55	1133.86	1<br>2<br>3<br>4	1134.87<br>567.94<br>378.96<br>284.47	1134.91<br>568.26<br>379.44<br>284.85	+4
**C1**	C8-RR-	L-Cystine diamide	1115.47	1114.67	1<br>2<br>3<br>4	1115.68<br>558.34<br>372.56<br>279.68	1115.64<br>558.26<br>372.10<br>279.76	+4
**C2**	C10-RR-	L-Cystine diamide	1171.57	1170.73	1<br>2<br>3<br>4	1171.74<br>586.37<br>391.25<br>293.69	1171.86<br>586.76<br>391.54<br>294.27	+4
**C3**	C12-RR-	L-Cystine diamide	1227.68	1226.79	1<br>2<br>3<br>4	1227.80<br>614.41<br>409.94<br>307.71	1227.85<br>614.94<br>410.26<br>-	+4
**C4**	C14-RR-	L-Cystine diamide	1283.78	1282.86	1<br>2<br>3<br>4	1283.86<br>642.44<br>428.63<br>321.72	-<br>642.51<br>428.76<br>-	+4
**C5**	C8-FRR-	L-Cystine diamide	1409.81	1408.81	1<br>2<br>3<br>4	1409.81<br>705.41<br>470.61<br>353.21	-<br>705.84<br>470.98<br>353.59	+4
**C6**	C8-NleRR-	L-Cystine diamide	1341.78	1340.84	1<br>2<br>3<br>4	1341.85<br>671.43<br>447.95<br>336.22	-<br>671.85<br>448.49<br>336.25	+4
**C7**[b]	C8(4)-RR-	L-Cystine diamide	1227.68	1226.79	1<br>2<br>3<br>4	1227.80<br>614.41<br>409.94<br>307.71	-<br>614.82<br>410.37<br>308.14	+4

a – ion charge; **b** – C8(4) – 2-butyloctanoic acid residue.

Table S2. Percentage of **B7** and **C7** remaining upon incubation with normal human serum.

Time (h)	B7 remaining (%)	C7 remaining (%)
0	100.0 ± 2.4	100.0 ± 1.1
1	94.3 ± 1.8	92.2 ± 2.1
2	87.3 ± 1.7	82.5 ± 1.3
3	83.1 ± 1.6	79.2 ± 1.6
4	80.1 ± 1.2	72.8 ± 3.0
5	63.2 ± 2.9	59.4 ± 1.0

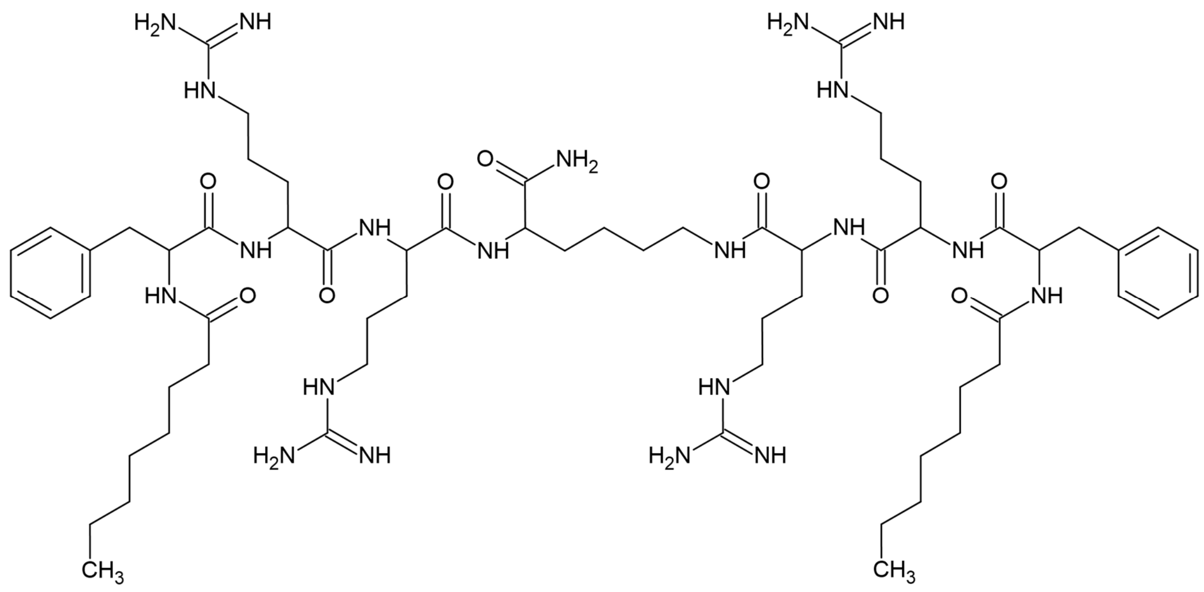

Figure S1. Chemical structure of **B5**.

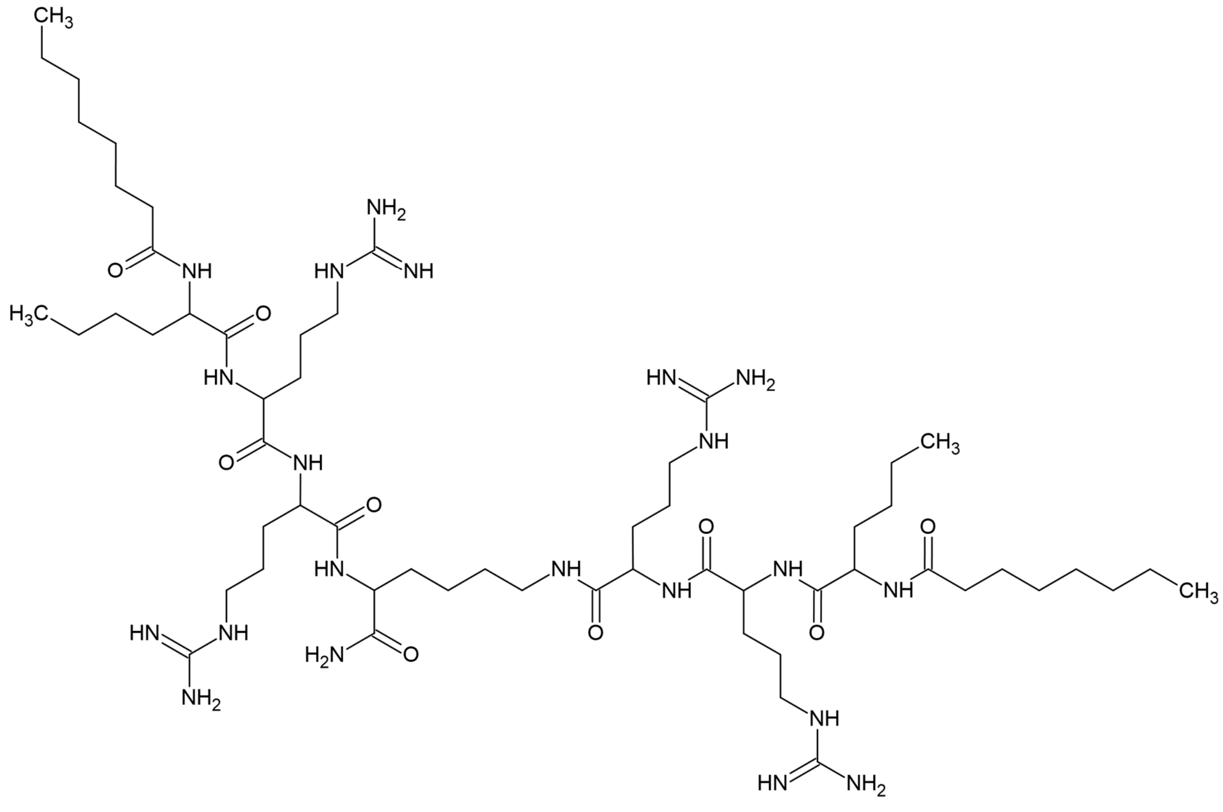

Figure S2. Chemical structure of **B6**.

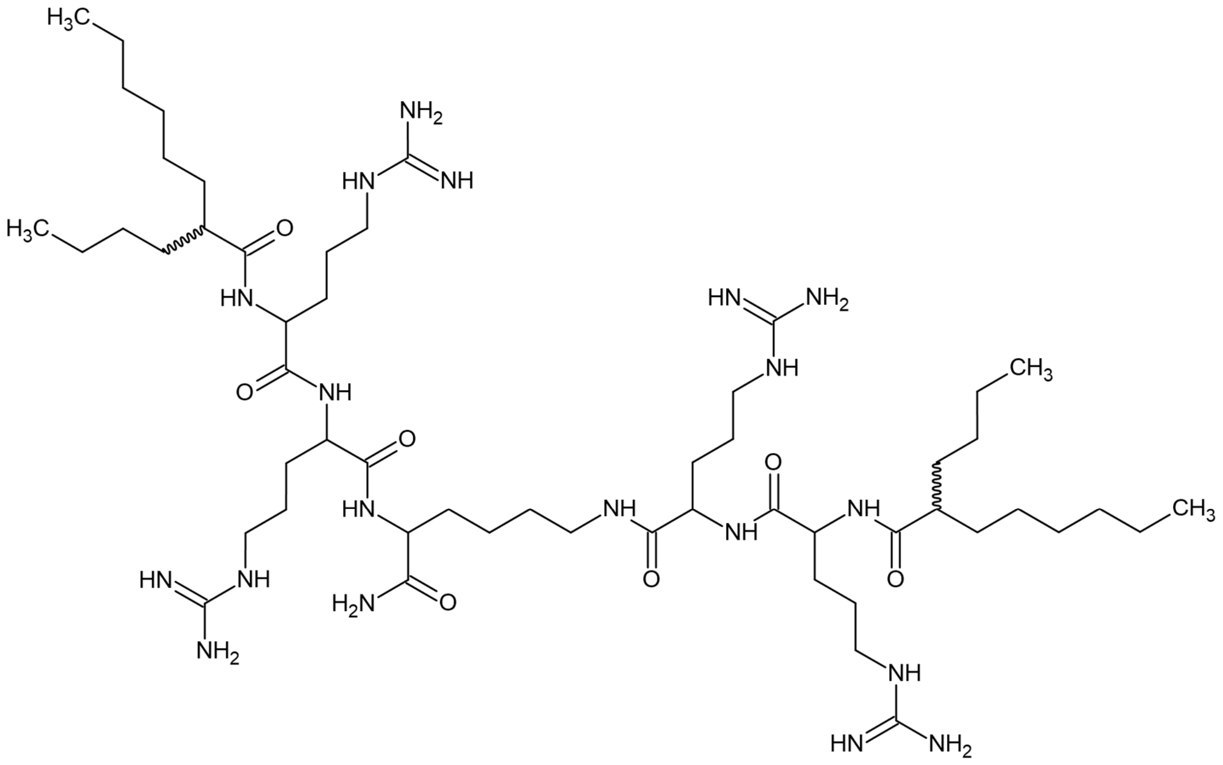

Figure S3. Chemical structure of **B7**.

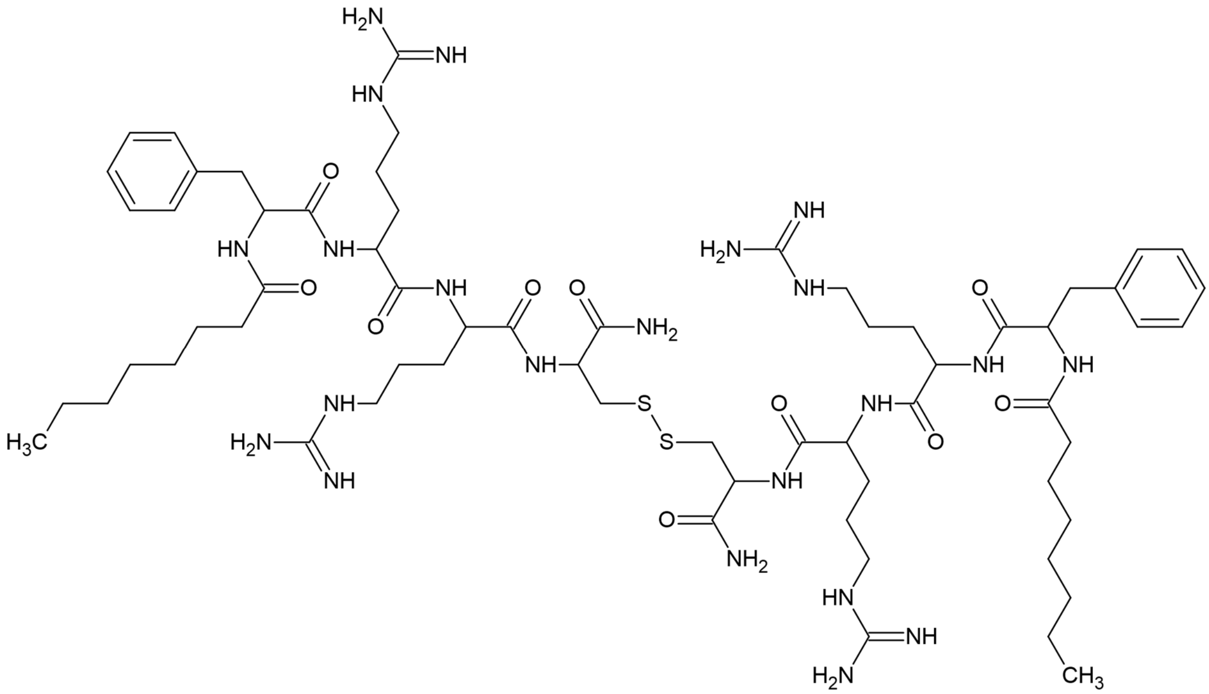

Figure S4. Chemical structure of **C5**.

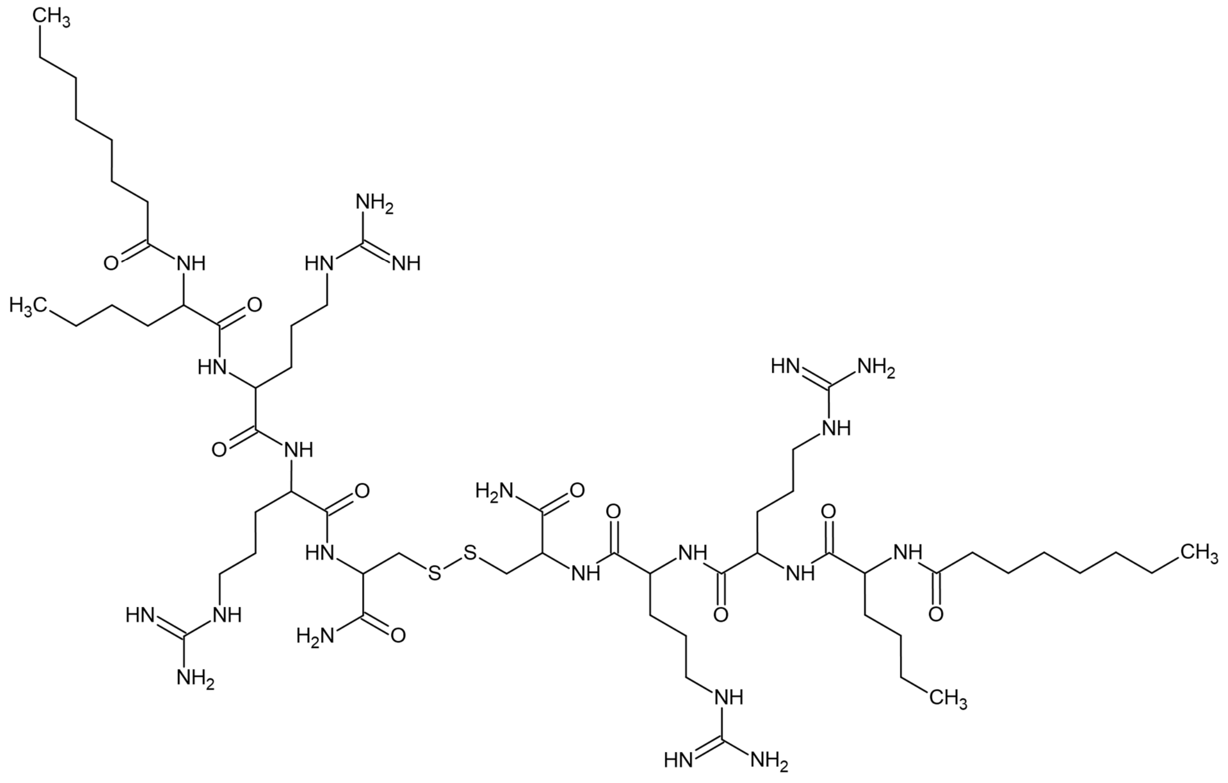

Figure S5. Chemical structure of **C6**.

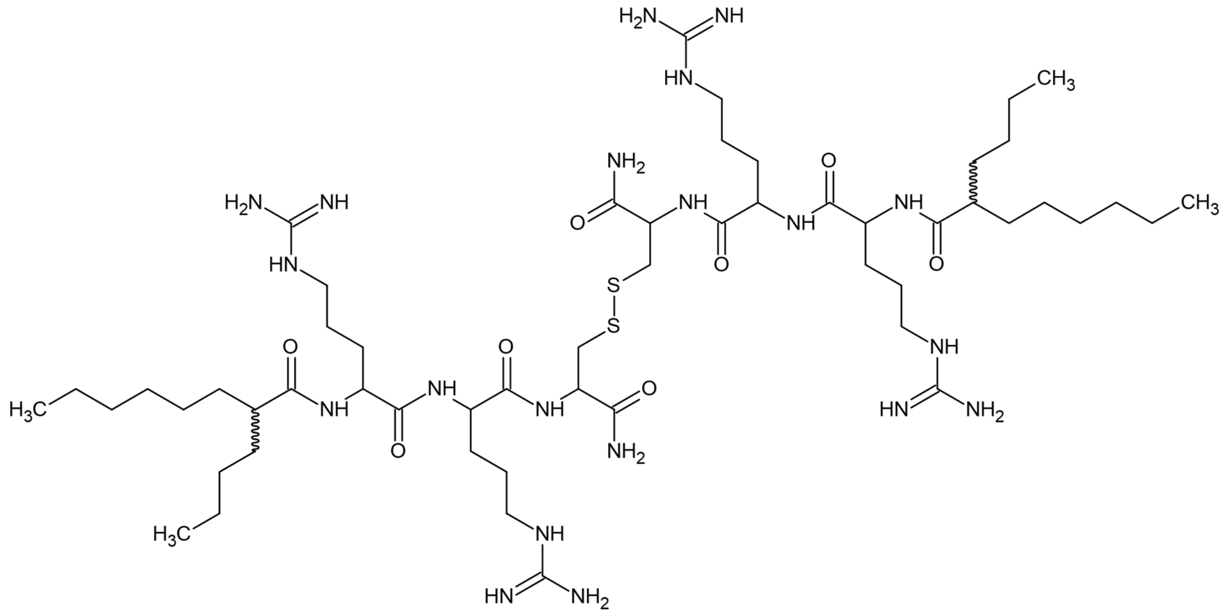

Figure S6. Chemical structure of **C7**.

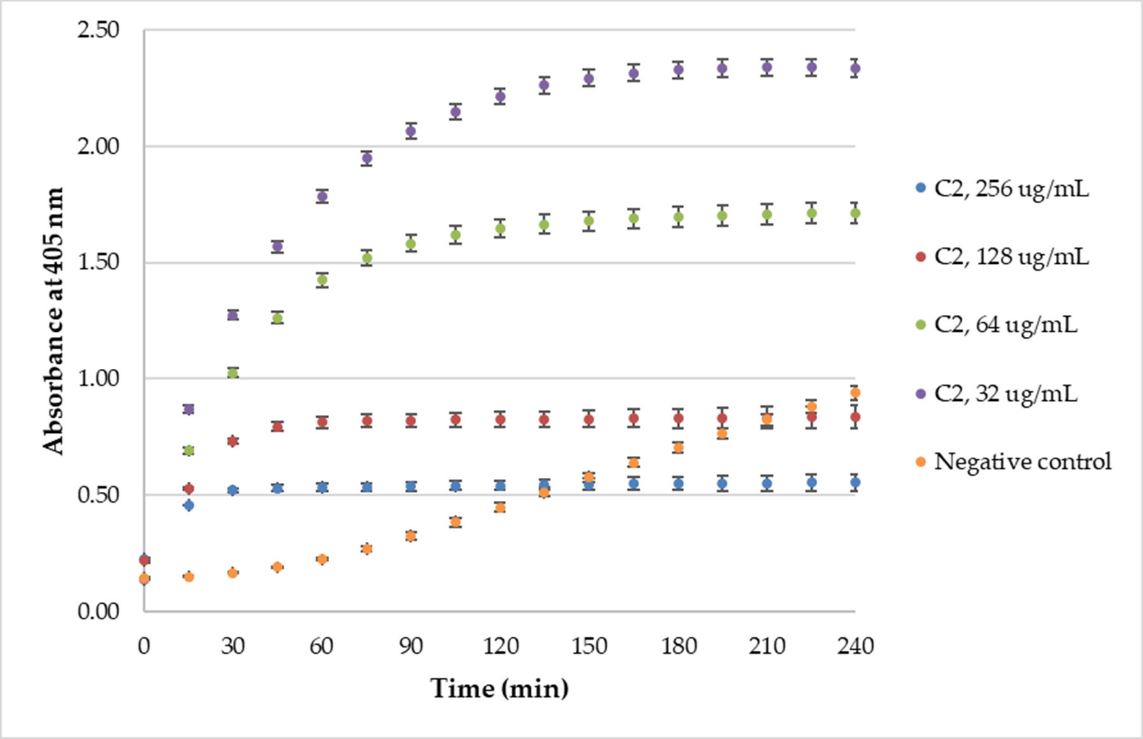

Figure S7. IM permeabilization kinetics of **C2**.

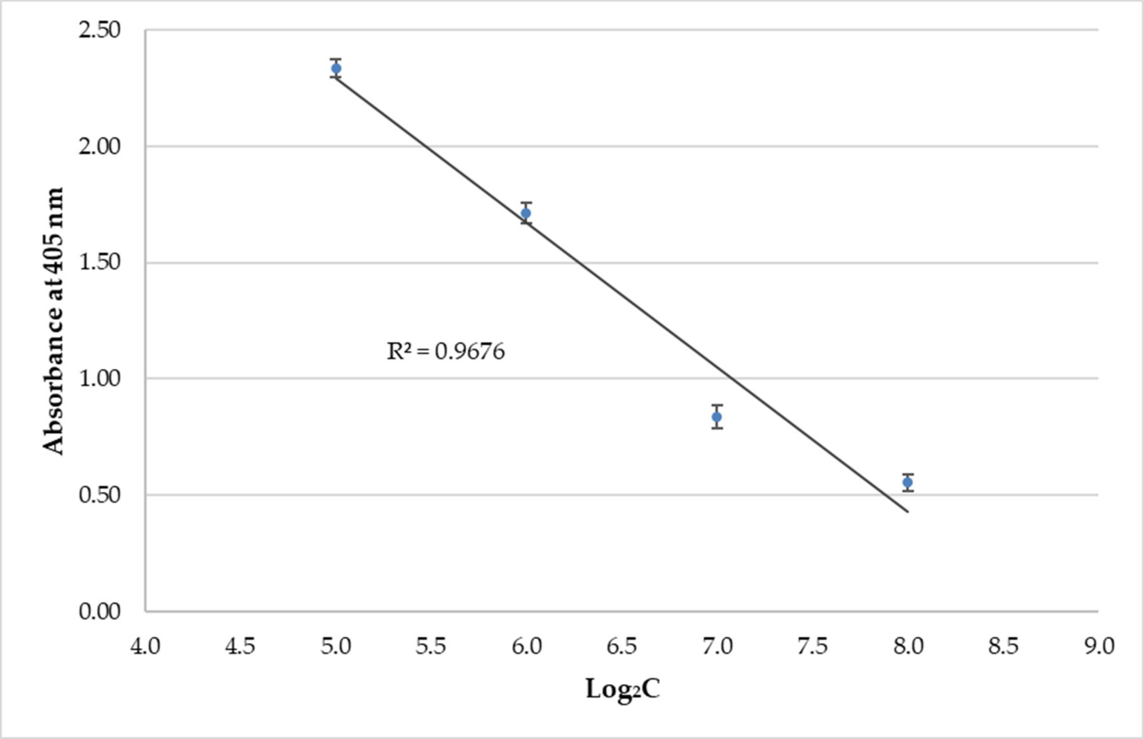

Figure S8. Log_2(**C2** concentration) vs. absorbance at 405 nm at 240 min.

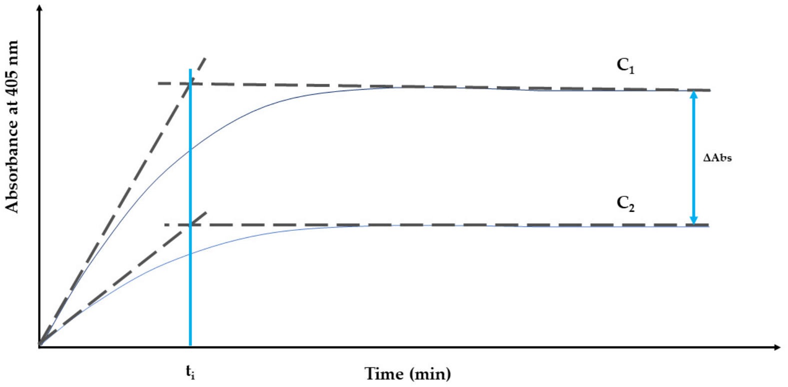

Figure S9. Exemplary results of membrane kinetics experiments.

Presumably, compounds at some concentrations can affect absorption of the product. Two compounds with identical reaction kinetics but different effect on absorption of the product (e.g. ONP) must have equal t_i. In fact, t_i seems to be a reasonable parameter that enables to compare kinetics due to changing the slope of the curve and different values of absorption maxima. This parameter (t_i) is analogous to $t_{1/2}$ determined by Sciacca et al in a study on the kinetics of dye leakage from large unilamellar vesicles. The $t_{1/2}$ is the time, half of which is defined as the time the signal needs to reach 50% of the maximum [1].

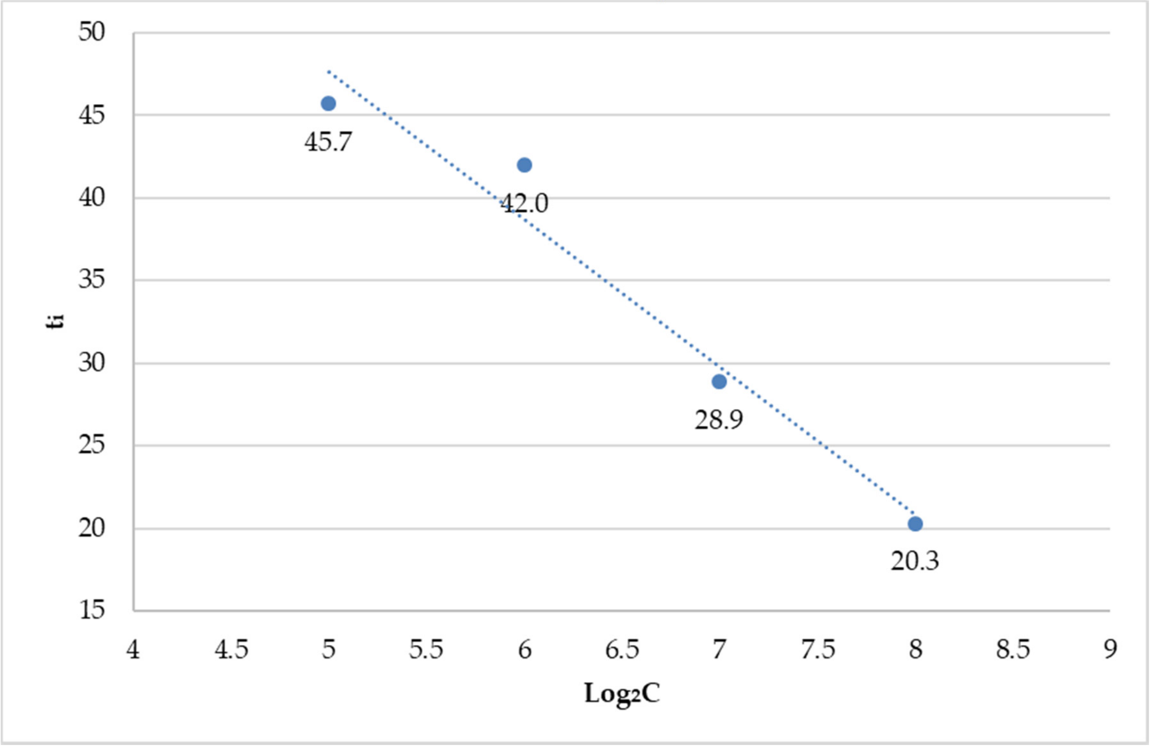

Figure S10. Log_2(**C2** concentration) vs. t_i at different concentrations of **C2**.

References

1. Sciacca, M.F.M.; Chillemi, R.; Sciuto, S.; Greco, V.; Messineo, C.; Kotler, S.A.; Lee, D.K.; Brender, J.R.; Ramamoorthy, A.; La Rosa, C.; et al. A blend of two resveratrol derivatives abolishes hIAPP amyloid growth and membrane damage. *Biochim. Biophys. Acta - Biomembr.* **2018**, *1860*, 1793–1802, doi:10.1016/j.bbamem.2018.03.012.
